# Abstracts of papers presented at the 1999 Pittsburgh Conference

**DOI:** 10.1155/S1463924699000206

**Published:** 1999


Journal of Automated Methods & Management in Chemistry, Vol. 21, No. 5 (September–October 1999) pp. 145–183

### Abstracts of papers presented at the 1999 Pittsburgh Conference

The following 104 abstracts form Part A of three issues of *Journal of Automated Methods & Management in Chemistry* devoted to abstracts of papers presented this year at the 50th Anniversary Pittcon held in March in New Orleans, LA. The editors have selected, from over 1000 presentations, those of particular interest to the Journal's readers. For information on next year's Pittcon, contact The Pittsburgh Conference, 300 Penn Center Boulevard, Suite 332, Pittsburgh, PA 15235–5503, USA. Tel.: + 1 412 825 3220; Fax.: + 1 412 825 3224; website—http://www.pittcon.org.

### Microfabrication as a route to nanovolume separation systems

*Fred E. Regnier, Brian Burke and Bing He, Department of Chemistry, Purdue University, W. Lafayette, IN 47907, USA*

The objective of the work described in this presentation is to provide a multidimensional analytical system for the analysis and characterization of proteins; particularly in complex mixtures. Detailed characterization of protein structure generally involves proteolysis and analysis of peptide cleavage fragments by reverse phase chromatography and/or capillary electrophoresis followed by mass spectrometry. Particularly in the case of protein regulation studies, large numbers of analyses are necessary. At issue is how to achieve high throughput in systems where chromatographic separations are necessary. Many believe that microfabricated, parallel-channel, microfluidic systems will play a role. This paper will explore reversed-phase separations of peptides in nanolitre volume, microfabricated columns. The fact that the phase ratio and detection path length are small in these systems is often considered to be a limitation. How to overcome these problems and even exploit them will be discussed.

### Fast FTIR imaging of multicomponent polymer systems

*J. L. Koenig and C. M. Snively, Department of Macromolecular Science, Case Western Reserve University, Cleveland, OH 44106, USA*

Fast FTIR imaging is a recent advancement in FTIR spectroscopy resulting from the coupling of a focal plane array (FPA) detector to an infrared microscope. This allows spatially resolved infrared spectral information to be acquired with high spatial and spectral resolution in a fraction of the time required by classical FTIR microspectroscopic techniques.

Due to the high spatial resolution and high chemical sensitivity of fast FTIR imaging, microscopically heterogeneous systems can now be studied in greater detail than ever before, resulting in a wealth of new information. We will present the application of this technique to the study of both phase-separated polymer blends and multicomponent semicrystalline polymer blends. The high chemical sensitivity of FTIR spectroscopy is used here as a contrast mechanism, which allows the micromorphology to be determined with great accuracy.

Additionally, due to the much reduced collection time associated with fast FTIR imaging, dynamic processes can be studied *in situ* and in real time. This approach has been applied to the study of diffusion processes in polymeric systems. We will show that, not only can the dynamic process be visualized, but that quantitative information can also be obtained, which is useful in fully describing the nature of the diffusion behaviour of the system.

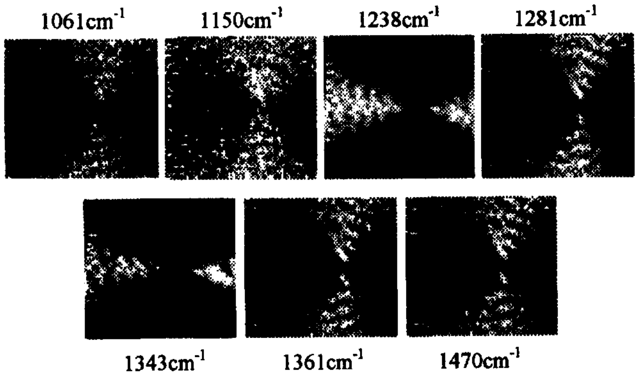

*Dichroic ratio images of the center of a poly(ethylene glycol) spherulite, genetated from different spectral bands, showing different relative strenghts of dichroism and different orientation of the transition moment with respect to the chain axis.*

### The emergence of PC-based measurement and automation at Pittcon

*Dudley D. Baker, National Instruments, 11500 N. Mopac, Expwy, Austin, TX 78759-3504, USA*

In the 1980s, all of the instrumentation systems at Pittcon were closed, proprietary systems that were difficult to integrate with other equipment. Moreover, they were expensive and virtually impossible to modify or upgrade. What the industry needed but did not have was a common platform for instrumentation. Few realized that the personal computer they were using to write their reports was destined to be that platform.

At Pittcon '87, National Instruments demonstrated a PC connected to an instrument, controlled with our LabVIEW software system. We demonstrated how you could build a 'virtual instrument' that would configure the instrument and then collect, analyse, present and store the data. In response to customer interest, we have brought back additional PC-based solutions to Pittcon exhibits, for plug-in data acquisition, image processing and motion control.

Pittcon is our mecca for learning about applications in chromatography and spectroscopy. For instance, we have learned that analytical chemists do not always have the expertize or the time to configure the system they need using our hardware and software tools. But fortunately, at Pittcon we have found third-party developers and integrators who have applied our products in fields, e.g. chromatography and chemometrics.

This year, almost every booth at Pittcon includes a PC. It may not even be visible, but it is there somewhere. Whether you are in the laboratory, the processing plant, or the field, you can use PC-based measurement and automation systems. At Pittcon, you can learn about the latest computer technologies, e.g. Windows NT, ActiveX and the Internet. You will see how PC measurement and automation delivers flexible, scalable systems that improve your productivity, get your products to market faster, and—most importantly—reduce your costs.

### The influence of the Pittsburgh Conference on Labtech

*Frederick A. Putnam, CEO, Laboratory Technologies Corporation, Two Dundee Park, Ste. B09, Andover, MA 01810, USA*

Our company was decidedly influenced by the Pittsburgh Conference. This influence actually began before the inception of the company in 1981. Attending several of the Pittsburgh Conferences in the 1970s as a graduate student was an important step in becoming familiar with the state of the art in instrumentation. Innovation in instrumentation was part of the research. Not only were we keeping up with what was available commercially, we were also always building new instruments in our own labs. These were 'homebrewed' many times because the research demanded instruments that were beyond (or just cheaper than) what was available commercially. Computer-based instrumentation in the 1960s and early 1970s was only available to labs with big budgets. Mainframes and minis were expensive. FTIRs were a notable example of mini-based instrumentation seen at Pittcon. Then microprocessors burst on the scene. They offered radically lower cost intelligence, and they were incorporated into 'homebrew' prototype-board instruments in the university labs we worked in at Carnegie-Mellon University and MIT. Soon, the first instruments based on mass-produced personal computers appeared at Pittcon. These used the Apple II. The race was on to develop PC-based instruments, test market ideas through papers and booths at Pittcon, and shape these into useful tools for science and engineering. The story of that race is full of many ups, downs and surprises in technology, business, science and showmanship.

### Lab automation: a Pittcon and ASTM synergy

*Robert Megargle, Emeritus, Cleveland State University, Cleveland, OH 44115, USA*

The advent of lab automation paralleled the development of small computers in the late 1960s. The effort was directed in part to automation of the measurement process, e.g. sample trays that moved specimens sequentially into an instrument pickup station, and thus provided unattended operation after an initial set-up. It was also directed at result processing where raw sensor data were changed into useful information in standard scientific form and units. Prior to computers, e.g. it was not uncommon to spend days converting the raw data from a half-hour GC/MS run into rationalized mass spectra plots needed for interpretation.

The early lab automation work was performed by chemists who saw the need, then honed their knowledge and skills of computers and electronics to get the job done. Today, professional engineers and computer specialists perform much of this work. The need for competent chemists has not been eliminated, however, because it is the chemist who understands better than anyone the nature and complexity of the problems to be solved.

Throughout this era, the Pittsburgh Conference was the premier scientific meeting and exhibition show of analytical chemistry, the area most impacted by lab automation. As such, the Pittsburgh Conference chronicled the improvements in automation, from the technical papers that reported advances and novel approaches, to the display of new instruments and apparatus on the exhibit floor.

In the 1970s, a far-sighted group, working under the leadership of Jack Frazier of Lawrence Livermore Labs, began a pioneering effort to define the automation process. The goal was to demystify the use of computers in the lab, help chemists learn to deal with computers effectively, and reduce the amount of rework and scrapped projects that were caused by poorly directed efforts. The group chose ASTM as a host for these efforts, but they met regularly at the yearly Pittsburgh Conferences. The committee produced a series of standards that are still in print today and provided a valuable training opportunity for the participants who exchanged knowledge and ideas at the meetings.

As an observer and participant in this monumental paradigm change in the way chemists perform their work, from manual analogue measurements to digital automated methods, the author is able to document the advances that took place, provide personal observations, and reflect on the improvements in productivity and scope of knowledge that were made possible by computer automation.

### Laboratory sample processing—making things go bump in the night

*Frank W. Plankey, Software Engineering Department, Zymark, Zymark Center, Hopkinton, MA 01748, USA*

The use of computers in the laboratory since the mid-1970s has forever changed the way that scientists work in their labs. The early use of computers to acquire data, process data, present data and provide for information in the lab was an essential first step in the laboratory computerization process. Later, the scope of this use was increased to include control of instruments, and the computer became part of the feedback and control loop with systems, e.g. the Fourier transform spectrometers. Computers could do things that humans either could not do or that were impractical for humans to perform. The benefits from this use of laboratory automation are enormous and the breadth of this type of activity increases every year. The real fun began when computers were challenged with doing things that humans could do; things that involved sample preparation and sample introduction into analytical instruments. Now all of a sudden, the computer was expected to be as capable as a real person who has the manual dexterity and pattern recognition needed to take a real sample, in its raw form and process it through the myriad of steps which are required to allow it to be introduced into a very finicky and complex instrument. These things included grinding, weighing, mixing, dispensing, diluting, decanting, centrifugation, filtering, heating, cooling and many other preparation steps. These are the manual steps which every laboratory scientist hates, and which require both the attention to detail and subtle pattern recognition that humans are able to provide but which machines cannot achieve.

This paper will look at some of the developments which have occurred in sample preparation using robotics to work up samples for analysis. We will explore how interactions with the intended audience at the Pittsburgh Conferences through the years has influenced the development of this technology. And, we will explore where we are today and where this field seems to be headed in the next few years.

### Microwave-assisted evaporation: re-evaluating the traditional chemistry of analyte recovery

*Dirk D. Link and H. M. 'Skip' Kingston, Duquesne University, Department of Chemistry and Biochemistry, 600 Forbes Ave., Pittsburgh, PA 15282-1503, USA*

Much of the chemistry of analyte dissolution and recovery documented in the literature has been written from the perspective of classical methods. Typical evaporation methods using conductive/convective heating mechanisms have been used to evaluate the temperature and the extent of analyte volatilization while heating a solution. With this type of heating, the walls and bottom of the vessel, which are directly contacting the heat source, continually become hotter throughout the evaporation process. As the sample size decreases, the sample may approach the temperature of the surface of the hot plate itself. As dryness is approached, sample temperatures are nearly impossible to control.

Microwave-assisted methods of sample processing have revolutionized the way that most analytical procedures can be performed. In contrast to typical heating methods, microwave energy involves unique heating mechanisms whereby aqueous samples interact with microwave energy to produce heat *in situ*. Use of microwave energy for dissolution of samples is well known. However, little work has been performed to investigate how these mechanisms may alter the evaporation process, which in turn may effect the volatility of analytes. A prototype apparatus has been designed to determine how the mechanistic differences involved with microwave heating give microwave-assisted evaporation advantages over traditional evaporation methods. Unlike current commercially available evaporation equipment, this novel apparatus provides the capability of monitoring and controlling several parameters (temperature, pressure, etc.) inside the vessel during the evaporation process.

As, Cr, Sn and Hg are among the analytes that are traditionally described in the literature as being volatile when heated. Losses of these analytes due to volatilization, especially as the chloride species, have been reported to be as high as 99% using traditional heating methods (e.g. hot plate). This work compares the volatility of several target analytes (As, Cr, Hg, etc.) during the evaporation process using both hot plate and microwave energy, and compares the fundamental parameters associated with analyte volatility using both methods. Due to the unique mechanisms of absorption of microwave energy by small aqueous samples, those analytes typically described as being volatile in the literature show greater recoveries when microwave energy is used as the heat source. Moreover, microwave heating allows more precise control of the conditions throughout the evaporation process, even as the sample approaches dryness. In light of the development of the microwave-assisted evaporation process, a re-evaluation of the classical literature pertaining to analyte volatility is needed.

### A novel on-line micro-extraction system of metal traces for their subsequent determination by plasma atomic emission spectrometry using pH-zone-refining high-speed countercurrent chromatography

*Eiichi Kitazume, Tadako Higashiyama, Nobuyoshi Sato, Masahumi Kanetomo[1], Takeshi Tajima[2], Seiichiro Kobayashi[2] and Yoichiro Ito[3], Faculty of Humanities and Social Sciences, Iwate University, Morioka, Iwate, 020-8550 Japan; [1]Central Research Laboratory, Hitachi, Kokubunji, Tokyo 185-8601, Japan; [2]Hitachi, Tokyo Electronics, Kokubunji, Tokyo 185-8601, Japan; [3]Laboratory of Biophysical Chemistry, National Heart, Lung, and Blood Institute, NIH, Bethesda, MD 20892, USA*

A highly efficient enrichment of metal ions was achieved by pH-peak-focusing high-speed countercurrent chromatography (CCC) that is developed by the variation of the pH-zone-refining high-speed CCC. The peak intensities for a 10-ml standard sample in the effluent stream were increased over 100-fold compared with the conventional plasma atomic emission spectrometry. In this method, Ca, Cd, Cu, Mg, Mn and Zn are chromatographically extracted in a basic organic stationary phase containing a complex-forming reagent, e.g. di(2-ethylhexyl) phosphoric acid by introducing the sample solution into the column rotated at 1200 rpm. When the column is eluted with the acidified mobile phase, metal ions are trapped and concentrated around the sharp pH border formed between the acidic and basic zones, moving toward the outlet of the column. Enriched metal ions are finally eluted with the sharp pH-border as a highly concentrated peak into a less than 100-μl volume. The capability of the

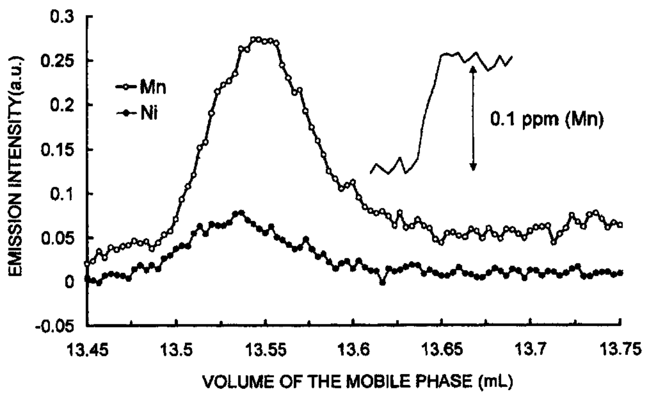

*Enrichment profiles of tap water. 10 ml of sample and 0.6 ml of the stationary phase were introduced into the column line immediately after revolution was started.*

present method is evaluated in terms of concentration efficiency and peak resolution of a target element from matrices in trace determination in tap water using various column diameters.

The figure shows an enrichment profile for Mn and Ni in 10 ml of tap water. The effluent from the outlet of the column was diluted 10 times with water just before entering into the detector so that the concentration profile of the metals in the effluent is more precisely traced.

### On-line monitoring of amino acid neurotransmitters *in vivo* by microdialysis with flow-gated capillary electrophoresis

*Steven R. Witowski and Robert T. Kennedy, Department of Chemistry, University of Florida, Gainesville, FL 32611-7200, USA*

Current separation-based methods of monitoring the neurotransmitter amino acids (glutamate, aspartate, glycine, GABA and taurine) *in vivo* involve three major steps: (i) collection of dialysate; (ii) derivitization of sample; and (iii) separation of desired analytes. When HPLC is used for separation, an off-line approach is typically used and sample collection is the limiting step with respect to temporal resolution due to a minimum required volume for reaction and injection. With CE an on-line method can be used thereby eliminating the collection step, making separation the limiting factor for temporal resolution. Our group has previously developed an on-line flow-gated CE system to monitor glutamate and aspartate every 5 s. Microdialysate is reacted with *o*-phthaldehyde/β-mercaptoethanol for a predetermined time, and ~27 pL is injected onto the separation capillary via a flow-gated interface. Zones are detected fluorescently using the 354 nm line of a He [illegible]d laser. Temporal resolution for this system was found to be between 7 and 14 s, and was in fact limited by the microdialysis probe itself and dead volume in the system up to the flow-gated interface.

We have been able to improve temporal resolution by the construction of a zero dead volume tee for the mixing of microdialysate with derivitization reagent. Under the same conditions as before, temporal resolution is now less than 4 s, making the separation the limiting step. This improvement has allowed us to use smaller microdialysis probes and lower flow rates while maintaining the required temporal resolution and relative recovery for *in vivo* measurements in previously inaccessible brain regions. For example, we have used 1 × 0.2 mm o.d. microdialysis probes at a flow rate of 250 nl/min in the rat hippocampus to monitor glutamate and aspartate with a 7-s temporal response and 40% relative recovery. In addition we have identified and monitored peaks corresponding to arginine, glycine, reduced glutathione and *o*-phosphoethanolamine in the electropherograms.

### Direct detection of DNA with an integrated detector on a microfluidic chip

*C. Brandon Davis, Matt McCormick and Werner G. Kuhr, Dept. of Chemistry, University of California, Riverside, CA 92521, USA*

A microfluidic chip has been developed which allows for the direct detection of several distinct DNA targets present in a sample. Utilizing a photoactivatable form of biotin, DNA probes can be attached in microchannels present on the chip via biotin avidin linkage. The sample is then introduced into the channel where an appropriate DNA target hybridizes with its complementary probe. Following hybridization of the target, an alkaline buffer is introduced to the channel to dehybridize the double-stranded DNA and flush the target downstream to a copper electrode. The dehybridized DNA is then detected using sinusoidal voltammetry. The elution time can be used to identify the particular DNA target as the DNA probes are spatially segregated in the channel. Integrating the detector and the sensing probes on the microfluidic chip allows for an inexpensive and easily fabricated biosensor device for the precise recognition and subsequent detection of a specific complimentary DNA target for diagnosis and genetic screening.

### High-speed gas chromatographic forensic screening for drugs of abuse

*Tricia A. Williams, Michelle B. Riddle, Stephen L. Morgan and William E. Brewer[1], Department of Chemistry & Biochemistry, The University of South Carolina, Columbia, SC 29208, USA; [1] Veterinary Diagnostic Laboratory, Clemson University, P.O. Box 102406, Columbia, SC 29224-2406, USA*

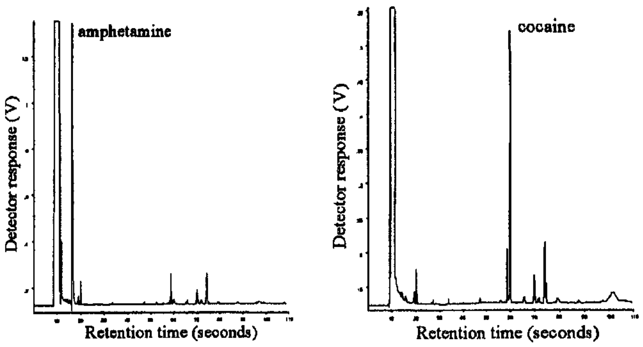

As courts have become backlogged in recent years with slow case turnaround, the development of faster routine screening methods for drugs of abuse is particularly significant. The availability of narrow-diameter, thin-film, short-capillary columns, and novel column/instrument designs capable of direct resistive column heating at rates up to 30 °C/s have stimulated use of gas chromatography for rapid analysis. In this paper we report the application of the Flash-GC™ (Thermedics Detection, Chelmsford, MA, USA) to perform extremely rapid gas chromatographic screening for drugs of forensic relevance. For example, a standard mixture of 17 of the most important drugs of abuse (amphetamine, methamphetamine, butabarbital, amobarbital, meperidine, pentobarbital, secobarbital, glutethimide, phencyclidine, methaqualone, methadone, cocaine, amitriptyline, imipramine, doxepin, desipramine, pentazocine, codeine and oxycodone) can be separated in under 70 s. A systematic investigation of the effects of programming rates and timing variables associated with Flash GC separations exhibited the traditional trade-off between analysis time and resolution. Case studies of confiscated street drugs containing amphetamine, cocaine and heroin were analysed to test the repeatability of retention times. Ten replicate injections over a 2-day period (five runs on the first day, followed by another five runs on the second day) achieved relative standard deviations for retention times in the range of 0.48–0.76%. These results demonstrate that Flash-GC$^{TM}$ is capable of reduced analysis time while maintaining excellent resolution and retention time reproducibility.

### Analysis of petroleum fuels by comprehensive two-dimensional gas chromatography with mass spectrometry detection (GC × GC/MS)

*Glenn S. Frysinger and Richard B. Gaines, Department of Science, US Coast Guard Academy, 27 Mohegan Avenue, New London, CT 06320-8101, USA*

Petroleum fuels are complex mixtures containing many thousands of chemical components. GC/MS methods are frequently used for separation and identification of these components, but numerous GC coelutions produce combined mass spectra where minor components can be masked. The limitations generated by the GC separation can be overcome by employing multidimensional chromatographic separations, e.g. comprehensive two-dimensional gas chromatography (GC × GC).

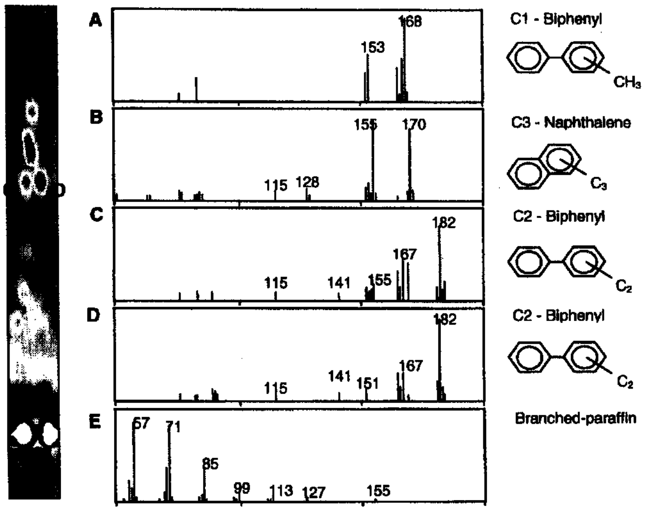

*Portion of petroleum GC × GC chromatogram demonstrating GC × GC separation, mass spectrometry detection, and chemical class identification.*

GC × GC uses two serial columns and a thermal modulator to produce highly separated and ordered chromatograms from petroleum fuels. GC × GC can resolve thousands of peaks in a two-dimensional retention time plane, and group them according to chemical classes, e.g. paraffins, olefins, naphthenes and aromatics. In this work, a mass spectrometer is coupled to the GC × GC to provide a three-dimensional separation that can identify the resolved peaks.

The operation of the GC × GC/MS system, and its application to the analysis of petroleum fuels, will be presented. Specifically, the separation and identification of minor components, homologous series and chemical classes in diesel fuel will be demonstrated.

### Oil spill source identification by comprehensive two-dimensional gas chromatography (GC × GC)

*Richard B. Gaines and Glenn S. Frysinger, Department of Science, US Coast Guard Academy, 27 Mohegan Avenue, New London, CT 06320-8101, USA*

Forensic analysis to determine the source of spilled petroleum hydrocarbons commonly employs techniques, e.g. high-resolution gas chromatography (HRGC) and gas chromatography with mass spectrometric detection (GC/MS). These methods usually target certain high molecular weight compounds, or biomarkers, which have proven resistant to change in the petroleum sample from weathering processes. However, light petroleum fuels, e.g. diesel fuel, have little or none of these biomarkers. Furthermore, gas chromatography does not possess the resolving power to identify and quantify minor petroleum components necessary to differentiate very similar samples.

This presentation describes the application of comprehensive two-dimensional gas chromatography (GC × GC) with flame ionization detection to oil spill source identification. An anonymous oil spill case from the US Coast Guard's Marine Safety Laboratories (MSL) was analysed by GC × GC, and compared with HRGC and GC/MS analyses conducted by the MSL. Analysis by GC × GC included both qualitative and quantitative comparison between one slightly weathered spill and two potential source samples. Several classes of components were used in the analysis, including paraffins, naphthenes and aromatics. The high resolving power of GC × GC was especially helpful in identifying compounds and compound classes to be used in the analysis. GC × GC analysis resulted in a match between the spill sample and one of the source samples, and was consistent with the MSL results.

GC × GC instrumentation and performance, as well as qualitative and quantitative comparisons between the spill and potential source samples will be presented and discussed.

### GC refinery gas analysis using thermal conductivity and one-flame ionization detectors

*Rollen Anderson, Fausto Pigozzo and Don Clay, ThermoQuest Corporation, 2215 Grand Avenue Parkway, Austin, TX 78728, USA*

Refinery gas is a blend of different gas streams containing saturated and unsaturated hydrocarbons as well as certain inorganic gases, including hydrogen. Providing good analytical data requires that all of the components be separated chromatographically so they can be quantitated in a precise and accurate manner.

There are a variety of methods for producing good analyses of refinery gas composition, but nonetheless there is a need to continually improve analytical precision and overall productivity. Using a GC equipped with an adequate number of valves and the proper columns, the analysis can be streamlined to make it a relatively simple task.

Of special concern to refinery gas analysis is the quantitation of hydrogen. This component is typically measured using a thermal conductivity detector operating with nitrogen carrier gas. This is done to get around the unacceptable response of hydrogen when measured in a helium stream. The alternative is to perform a separate hydrogen analysis using a thermal conductivity detector running with nitrogen carrier and then measure the other inert gases with helium carrier. Using properly dosed helium, the second TCD can be eliminated and the analysis completed with one TCD and one FID.

In addition to the analytical hardware, it is also helpful to have the ability to report data in a single analytical chromatogram. With the proper signal switching capability, the output of the two detectors can be plotted on a single chromatogram, and the entire analysis, including data reduction, laid out in one report.

It is the purpose of this paper to describe an analytical system that will accomplish the needs of refinery gas analysis. The author will discuss the hardware required to provide sample introduction, separation and detection, and a data system that can report results in a simple yet useful form.

### 'SSTS-Ajax' for analysis on-line by gas chromatography

*Yingtao Yu and Gang Chu, Department of Applied Chemistry, Fushun Petroleum Institute, Liaoning, 113001, China*

A switching system of triplet samples (SSTS-Ajax) is introduced in this paper, which can be of great use for the analysis on-line by gas chromatography. By using SSTS-Ajax, the triplet samples (i.e. a sample and its two duplicates) can be obtained on-line at the same time and analysed one by one.

If the analysis of one sample is unsuccessful or abnormal, the duplicates can be employed in further analyses, without the following analyses on-line intermitted. Of course, it is convenient for the analyst to adjust the instrument used and to examine the stability and repeatability.

Alternatively, the quantity of one sample can differ from that of its duplicates by using sampling tubes of different size, respectively. Meanwhile, the combination of any two tubes can also be used in constructing a calibration curve.

Furthermore, a triplet sample of different quantity can be switched into each column in a gas chromatograph and analysed under different operating conditions, respectively. Hence, more information about the sample can be obtained. When one operating condition is not efficient enough to analyse all the components in a sample, SSTS-Ajax can be used to solve this problem to some extent.

Moreover, the switching triplet samples from different points on-line can also be obtained and analysed in turn. If desired, SSTS-Ajax can be developed further, with the function enhanced.

In principle, SSTS-Ajax consists of several ploy-way valves and sampling tubes. It is easy to operate and very helpful for analysis on-line by gas chromatography. The principles and usage of SSTS-Ajax will be described in detail in this paper.

### Batch and continuous flow reactors for benchtop microwave-accelerated organic synthesis

*Kenneth Borowksi and Camillo Piroia, Milestone, 160B Shelton Road, Monroe, CT 06468, USA*

The use of domestic microwave ovens in conjunction with pressure vessels to accelerate organic reactions was first reported in 1986. It was clear that microwave energy, combined with pressure, exponentially increased reaction rates. Microwave-assisted organic chemistry has received dramatic exposure in the last decade. Today, there are ~220 published articles and reviews. Even with this intense interest, the lack of suitable equipment has severely curtailed progress in exploring the benefits of microwave-assisted synthesis.

A new microwave system for organic synthesis needed to be designed 'from the ground up' specifically for use in laboratory conditions. Facilities needed to be created for the addition of reagents, the withdrawal of samples, magnetic stirring, post-reaction cooling, and the precise control of pressure and temperature. High-capacity, chemically inert reaction vessels had to be created to withstand the high pressure and temperatures generated.

A new line of products to promote microwave-accelerated organic synthesis have been introduced. Based on a revolutionary microwave labstation platform, a series of unique high-performance open, closed batch, and continuous flow reactors deliver accelerated reaction rates.

Microwave-accelerated reactions and reaction rates will be presented and reviewed in this paper.

### Integrated microwave extraction: a replacement for Soxhlet

*Robert C. Richter, Sejal Shah, George Lusnak, Dengwei Huo, H. M. 'Skip' Kingston and Camillo Pirola*[1]*, Duquesne University, Department of Chemistry and Biochemistry, 600 Forbes Ave.,*

*Pittsburgh PA 15282-1530, USA;* [1]*Milestone s.r.1, Via Fatebenefratelli, 1/5 24010 Sorisole (BG), Italy*

Organic extractions using Soxhlet methodology have been the norm for over 150 years. Soxhlet extraction often requires the sample to be dried before extraction and filtered after extraction. The extracted samples must also be concentrated or reconstituted in an appropriate solvent for analysis. This whole process requires several pieces of equipment and takes several hours to complete. In integrated microwave extraction, the same piece of equipment is used for drying, extracting, filtering and evaporation. This design minimizes sample manipulation leading to a semi-automated process. Another unique feature of this technology is that both polar and nonpolar solvents can be used for extraction. This allows the tailoring of the extraction process to specific compounds of interest. We have developed a procedure, using this technology, for the extraction of polyaromatic hydrocarbons (PAHs) from soils. Recoveries were equal to or better than Soxhlet, required less time than Soxhlet, and reduced the amount of solvent because the evaporated solvents are collected and recycled.

This technology is not limited only to organic extractions. We have successfully used integrated microwave extraction for the species extraction of Cr(VI) from soil samples. Supporting data for this application will also be presented in this paper.

### Rapid concentration of reagents by microwave heating under clean room conditions for ultratrace analysis

*Mike Moses and Gus Obleada, CEM Corporation, PO Box 200, Matthews, NC 28106, USA*

Manufacturers of semiconductor components and their reagent suppliers have a high requirement for ultra-trace analysis under clean room conditions. However, the sample preparation step for the analysis can often introduce more impurities than were present in the original sample. This effectively raises the sample detection limit above the levels that could be achieved by the analyser.

The conventional sample preparation process for these reagents, acids and solvents, involves evaporation to increase the concentration of the analytes of interest. The techniques of evaporating these acids and solvent mixtures involve slow, laborious hot plate steps. The use of microwave technology in a sealed environment allows reagents to be rapidly evaporated in a clean, controlled environment. This paper will discuss the microwave instrumentation used for the clean room evaporation step, and provide evaporation rate information and elemental recoveries for acid and solvent mixtures pertinent to the semiconductor industry.

### Combinatorial library compound characterization using GC-AED

*Cynthia Cai, Bruce D. Quimby and Randall Munson*[1]*, Hewlett-Packard, 2850 Centerville Road, Wilmington, DE 19808, USA;* [1]*Hoechst Marion Roussel, P.O. Box 6800, Bridgewater, NJ 08807, USA*

In the fast-evolving field of combinatorial chemistry, the number of compounds being synthesized by individual chemists has increased exponentially. In many cases, however, there are now bottlenecks that limit evaluation of these new compounds as to their potential usefulness. These bottlenecks include limited ability to perform high-throughput analysis, purification and data management for large libraries of synthesized compounds. The major challenges in high-throughput analysis are determining the identity, purity and amount (yield) of each synthesized compound. A further challenge is that these analyses must be performed with the least amount of sample consumption possible.

Atomic emission detection (AED) coupled with gas chromatography enables the chemist to quickly verify compound identity and purity while consuming only micrograms of the compound. GC-AED has the capability of measuring 23 different elements, including a few stable isotopes. Elemental response factors are independent of compound structure. This allows the chemist to measure the yield, purity and atomic ratios of discrete compounds or compound mixtures without the need for sample-to-sample calibration. The results of several experiments, including libraries generated on an automatic solution-phase synthesizer, will be presented in this paper. Techniques that extend the range of compounds that can be measured by GC will also be discussed.

### Second generation on-line SPE-LC, SPE-GC and SPE-MS

*J. A. Ooms, O. Halmingh, G. J. M. Van Gils and G. S. J. Haak, Spark Holland B.V, P.O. Box 388, 7800 AJ, Emmen, The Netherlands*

Solid phase extraction with direct elution into a HPLC, GC or (LC)-MS (on-line or hyphenated SPE) is a proven technology which is increasingly used to automate analytical assays including sample clean-up. Hyphenated SPE provides full automation of the entire assay and high precision and sensitivity because the extract is quantitatively transferred to the analyser without dilution. Although such benefits are highly appreciated, new developments and trends in the analytical laboratory are now demanding for significant improvements of the current technology.

The pharmaceutical industry, under strong pressure to increase the sample throughput, has reduced bioanalytical run times for HPLC and LC-MS significantly (typically 2–5 min). Consequently, equally high demands are made not only with respect to speed of SPE but especially with respect to the speed of method development for new on-line SPE assays.

SPE-GC for environmental analysis is very promising, but reliable routine operation requires a dedicated instrument configuration with a robust large-volume injection interface and PC control for the entire system configuration.

In this paper we present dedicated system configurations based on our PROSPEKT technology for rapid automated SPE-LC/MS method development, Highthroughput SPE-LC/MS analysis and robust SPE-LVI-GC/MS use the OPTIC-II as LVI interface. Applications for all systems will be shown.

In addition, some new SPE sorbents are evaluated with respect to speed of SPE and applicability as generic sorbent for fast method development of drug analysis assays.

### Automatic NMR, MS and IR spectra-structure compatibility tests for combinatorial chemistry and spectroscopic library integrity

*Renate Buergin Schaller, Patrick Fontana, Hans E. Holzgang, Bernhard Seebass, Erno Pretsch, Guenther Bovermann[1], Joaquim N. Figueiredo[1] and Hans Widmer, Department of Organic Chemistry, Swiss Federal Institute of Technology (ETH), Universitatstrasse 16, CH-8092 Zurich, Switzerland; [1]Novartis Pharma AG, CH-4002 Basel, Switzerland*

One of today's challenges in spectroscopic analysis is the need for increasing efficiency in solving spectra–structure issues. In many cases, a proposed structure is available in electronic form along with the measured spectrum so that the only question to be answered is whether the two agree or not. Especially in combinatorial chemistry, only yes/maybe/no (green/yellow/red) answers are sought. Recently, we have developed software modules for undertaking this task. The different approaches for IR, MS, $^{1}$H-NMR and $^{13}$C-NMR spectra have in common that, on the basis of the proposed structure, spectral properties are predicted and compared with the respective values automatically obtained from the spectrum. In this talk, the basis of these approaches is presented together with first results from an industrial analytical laboratory. The same approaches are applicable to screen already existing spectroscopic databases and as entry control for newly added data.

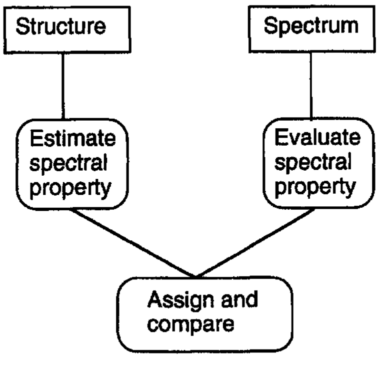

*Scheme of the spectra–structure compatibility test procedures.*

### Ultra-fast analytical and preparative HPLC for combinatorial chemistry and other applications

*Robert D. Ricker, John W. Henderson and Brian A. Bidlingmeyer, Hewlett-Packard, Little Falls Analytical Division-Newport, 538 First State Blvd, Newport, DE 19804-3552, USA*

Demand for better, faster and more economical analyses has brought about the development of smaller particles and column dimensions in liquid chromatography. Manufacturers now market columns with particle sizes under 5 μm, and columns with dimensions up to 10 times smaller than traditional analytical-size columns. While these column configurations have been possible for several years, their acceptance by chromatographers, in addition to improvements in instrument electronics and hardware, was needed to stimulate their widespread use. As an offshoot of the need for faster separations, preparative-scale (1″ diameter) short columns have been developed, allowing rapid separations with larger sample loads. The trend toward smaller column configurations (=1 mm id columns and 1.5 μm particles) continues, but practical considerations may limit the chromatographer's ability to obtain expected results for a particular column configuration and sample. Many of these issues will be addressed within the context of ultra-fast separations (e.g. <1 min) using current 3.5 and 5 μm totally porous silica technology. Where appropriate, theoretical and actual results will be contrasted, as well as possible explanations for differences between the two. Finally, 'ultra-fast' separations (e.g. <10 min) will be shown for the analysis of very complex samples (where 1 h would be a traditional run time).

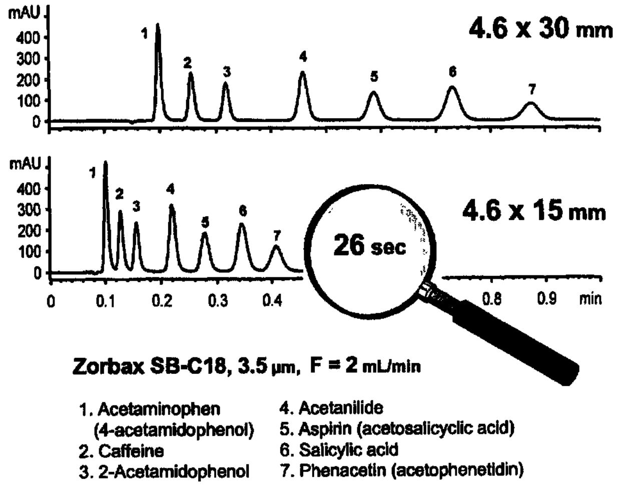

*Ultra-fast analytical separations at 70 °C.*

### A remote reflectance probe for determining the cleanliness of reactor walls: a potential alternative to clean and swab methods

*Mark A. Druy and Roy A. Bolduc, Sensiv, 195 Bear Hill Road, Waltham, MA 02451, USA*

In pharmaceutical manufacturing, it is critical to determine the cleanliness of the reactor walls prior to the blending/formulation process. The FDA requires pharmaceutical companies to 'prove' that the cleaning of mixing equipment not only removes all traces of the substances mixed in the container, but that the cleaning procedure does not introduce any foreign substances to the equipment. Current methods that are used to 'prove' the cleanliness of pharmaceutical equipment are based on HPLC, UV-Vis, etc. These methods are time consuming as they rely on first 'swabbing' a given portion of the inside surface of the blender with a cotton swab wet with an appropriate solvent before the analytical procedure is performed. We have developed a family of remote reflectance probes that eliminate the need to 'swab' the surface. Instead, a probe is used to directly examine the surface of the vessel. It utilizes the principals of infrared spectroscopy and it works in the mid-infrared region, as the ability to obtain spectral information in the mid-infrared region not only enables this probe to monitor processes, e.g. reactor cleaning, but also to identify contaminants based on their spectral fingerprints. This paper will discuss the design of this family of probes and their potential to be used for validating cleanliness of pharmaceutical vessels.

### Rapid determination of plasticizers in PVC, hexane extractables in polyethylene, and oils in rubber using accelerated solvent extraction

*Richard E. Carlson, Eric S. Francis, John L. Ezzell and Robert J. Joyce, Dionex Corporation, Salt Lake City Technical Center, 1515 W. 2200 S. Suite A, Salt Lake City, UT 84119, USA*

Accelerated solvent extraction (ASE®) uses pressurized organic solvents at elevated temperatures to increase extraction speed and efficiency. For many matrices and for a variety of solutes, ASE has proven to be equivalent or superior to Soxhlet and reflux extraction techniques.

Soxhlet and reflux extraction methods are time consuming and require large volumes of solvents. Accelerated solvent extraction methods typically range from 10 to 25 min in duration and require from 15 to 50 ml of extraction solvent, depending upon the sample size. The extraction of plasticizers, e.g. dioctyl adipate (DOA), dioctyl phthalate (DOP), trioctyl phosphate (TOP) and trioctyl trimellitate (TOTM) from poly (vinyl chloride) (PVC) is typically accomplished using a Soxhlet apparatus. For example, ASTM method D 2124 specifies a 6-h Soxhlet extraction. Likewise, the removal of hexane extractable from polyolefins (ASTM method D 5227) requires a 2-h reflux method. Current extraction methods for oils from rubbers, e.g. styrene-butadiene and ethylene-propylene-diene are also time consuming and require large volumes of extraction solvent.

Using ASE, the extraction of plasticizers from PVC is completed in ~12 min using only 25 ml of solvent. Similarly, accelerated solvent extraction has also proven to be effective in the extraction of the hexane-soluble fraction in polyolefins, and the removal of aromatic processing oils from rubber. This paper will detail the sample preparation and method development steps for the ASE extraction of additives from PVC, polyolefins and rubbers.

### Analytical instrumentation for the next millennium

*Jean H. Futrell, Department of Chemistry and Biochemistry, University of Delaware, Newark, DE 19716, USA*

This symposium is the first report of a workshop intended to pinpoint recent developments in analytical instrumentation, and generate a broad outline of needs and opportunities for instrumentation development in the next decade. Representatives of the chemical, biological and materials science communities, academic, industrial and national laboratory scientists, and instrument manufacturers have attempted to step back from extrapolations of current technology, and examine needs and opportunities which are at the interface of chemistry and biology. The implications of high-throughput analyses, real-time feedback and informatics driven by the interface of chemical measurements with combinatorial chemistry, genomic science and nanomaterials technology were considered, The drivers for next generation holistic instrumentation, computational and instrumental advances requirements and technology transfer strategies to enable their development were discussed. Principal conclusions and recommendations will be summarized in this paper.

### Enhanced elemental speciation through use of a switched plasma source and time-of-flight mass spectrometry

*Gary M. Hieftje, John P. Guzowski Jr and Jose A. C. Broekaert[1], Department of Chemistry, Indiana University, Bloomington, IN 47405, USA; [1]Institut fur Analytische Chemie, University of Leipzig, Linnéstrasse 3, D-04103 Leipzig, Germany*

Despite heroic efforts to the contrary, atomic spectrometry is not inherently suited to speciation analysis. In an atomic spectrometric technique, one of the main goals is to decompose a sample into its constituent atoms, which can then be analysed by means of techniques, e.g. atomic absorption spectrometry, atomic emission spectrometry and atomic mass spectrometry. Regrettably, in the process of decomposing the sample, information about the chemical form in which the elements were originally found is completely destroyed. As a result, speciation in atomic spectrometry is usually achieved by some preliminary sample-treatment technique. The most common techniques are chromatography or capillary electrophoresis. Yet, such methods are time consuming and cannot be used when the species to be determined are labile. In addition, such techniques depend upon elution time to identify the species of interest. Because elution times can change with chromatographic conditions and even with the properties of the sample (especially in capillary electrophoresis), species identification is often uncertain.

In this presentation, an alternative approach to speciation will be discussed. The approach relies upon an ion source that is capable of being operated at several different internal energy levels. At the highest energy setting, the source produces a simple atomic mass spectrum. Such a spectrum not only enables empirical formulae to be determined, but offers relatively uncluttered spectra and less ambiguous isotopic information. Furthermore, because many atomic ions (especially of carbon) can be produced from a single organic molecule, sensitivity in this mode is higher than is ordinarily encountered in fragmentation mass spectrometry. In the second mode of operation, the source produces a fragmentation mass spectrum, similar to what is ordinarily encountered in electron-impact ionization. This mode permits established spectral files to be searched to aid in the identification of an unknown molecule. In the third mode of operation, the source yields only a molecular ion, from which can be deduced the molecular weight of the unknown species. The three modes of operation, achieved in rapid succession, can then be applied to unknown molecules for a more unambiguous identification. Also, because the source is coupled to a time-of-flight mass spectrometer, the information can be obtained even on a chromatographic time scale, enabling this new and powerful speciation technique to be coupled with traditional ones.

### Managing the modern university analysis and instrumentation laboratory

*Thomas Lyttle, 3 Coiony Trail, Mandeville, LA 70448, USA*

Shared-use analytical instrumentation facilities are becoming increasingly necessary for universities to effectively invest their research dollars. As budgets tighten and funding becomes increasingly difficult to obtain/maintain, the university-wide facility can be a cost-effective research resource. This paper will describe a model, centralized instrumentation facility (CIF): the historical perspective, organization and function, funding sources, and rate and recharge considerations.

Most academic analytical instrument service facilities are both located and operated within a single department (chemistry, biochemistry, biology, etc.), or a single college (engineering, sciences, veterinary medicine, etc.). In some cases, several departments utilize these facilities. Typically, the departments or the dean of the college in which the facility is located heavily subsidizes these facilities. These types of subsidies usually include line-item salaries and an operating expense budget. In one study, the conclusion was that the average DNA facility required at least a 50% subsidy. This paper covers the author's vision of a truly university-wide facility that has been proven to work.

### Becoming a better analytical lab manager

*David W. Green, Analytical Chemistry Laboratory, Chemical Technology Division, Argonne National Laboratory, Building 205, 9700 S. Cass Avenue, Argonne, IL 60439, USA*

Many scientists, at some point in their career, can expect to be assigned responsibility for completion of a project that has schedules, budgets and several project team members. These scientists will likely draw upon their technical skills, but will also require management skills to do their jobs effectively. Although some organizations provide access to management training resources, the skills required (e.g. teamwork, negotiation) can be contrary to much of their scientific training (e.g. independence, certainty). Furthermore, not all organizations recognize the need to provide managers with assistance in acquiring management skills- a manager is on his/her own.

Some scientists move from management of small projects to the management of large projects or to positions requiring significant line-management responsibilities. The skills required to be a successful manager include many that come from the formal training and professional experiences of scientists. However, these skills alone are insufficient. New skills must be developed to be successful, including skills in areas where many managers have had little formal training.

The management of an analytical laboratory has much in common with management of other organizations. However, success in managing individuals from whom independence and creativity are expected cannot be achieved with approaches used for most other kinds of organizations. The ability of an analytical laboratory manager to develop required skills and be successful as a manager depends heavily on a recognition of the need for growth, access to suitable resources and a commitment to develop new skills. This paper will address alternative ways to identify and develop required management skills that allow analytical laboratory managers to do a better job.

### Sample preparation of small biological samples for analysis by ICPMS. Micro-samples by microwave

*Brian Buckley, Kevin Cashman, Testeroy Emerson and Willie Johnson, Environmental and Occupational Health Sciences Institute, Rutgers University, 170 Frelinghuysen Road, Piscataway, NJ 08750, USA*

Biological sample analysis for metals usually requires small sample handling techniques because the quantity of material for analysis is limited. Biopsies, tissues from small animals and culture experiments all produce samples in the tens of milligrams or less range. In addition to the small sample size, the ambient concentrations of these metal contaminants make detection more difficult even with the more sensitive analytical methods. The key is to keep the sample contained, manipulate the sample as little as possible and minimize the contamination so that small dilutions of the prepared sample provide accurate results. Sample containment can become difficult with samples containing high concentrations of fat. The large evolution of $CO_2$ and small sample size makes closed vessel preparation difficult. In order to perform analysis of metals by ICPMS on biological samples, methods must be developed that can follow all of the aforementioned constraints and contain volatile inorganics, e.g. Hg and As. Recovery measurement and quality control become difficult because there are no real reference materials that are homogeneous at low sample masses. Microwave sample digestion has been the answer for preparation of these small samples using one-vessel preparation schemes and micro-sample handling skills, but the question of suitable quality control remains.

This paper will focus on the sample preparation and analysis methods used on different biological sample types prepared for ICPMS analysis. Protein precipitation, animal tissue and human samples will be among the matrices digested and analysed by the microwave sample preparation and ICPMS systems. The performance of the digestion techniques on stable isotope labelling experiments for the differential uptake of mercury in the brain will be a highlight of this presentation. Successful experiments with laboratory animals demonstrated that these stable isotopes make excellent markers for mercury species labelling provided the correct digest procedure can be employed. The micro-samples generated with brain fragments from the mouse require micro-sample handling.

With each matrix a different set of problems and special needs arises, high fat for brain, high dissolved solids for blood, difficult digest conditions for hair, etc. These various special conditions required for each matrix type will also be examined. Special analysis protocols including cool plasma experiments for the measurement of easily ionized elements (EIEs) may also be presented.

### On-line capillary NMR detection for electrophoretic separations

*Michael E. Lacey, Zhixin J. Tan, Dean L. Olson and Jonathan V. Sweedler, Department of Chemistry, University of Illinois, Urbana, IL 61801, USA*

Analytical challenges presented by complex, mass-limited samples have driven analyses toward high-efficiency, microscale separation schemes, e.g. capillary electrophoresis (CE). While the high structural information content of NMR spectroscopy makes it attractive as a chemically rich detection mode, traditionally poor sensitivity has precluded its use as a detector for capillary separations. The development of microcoil probes with enhanced mass sensitivity allows the combination of CE with NMR detection. Fabrication of a solenoidal microcoil directly on a separation capillary creates an on-line, nanolitre-volume detector for CE. We demonstrate high-resolution proton NMR spectra at 300 MHz from picomole quantities. A detailed explanation of this phenomenon will be discussed along with alternative CE/NMR acquisition approaches to eliminate these effects.

Because of the advantages of greater spectral dispersion and increased sensitivity at higher magnetic fields, the development of probes for proton NMR at 500 MHz is presented. Furthermore, the coupling of these probes to CE separation of carbohydrates is explored. Carbohydrate separations have been achieved via complexation with borate. While the NMR spectra of carbohydrates in the presence of borate indicate complexation, the carbohydrates remain spectrally distinct. Because NMR provides data on complexation and structure, it is ideally suited as a detector for such difficult-to-observe analytes. The use of NMR not only as a detector but to provide diagnostic information about the CE separation conditions will be presented in this paper.

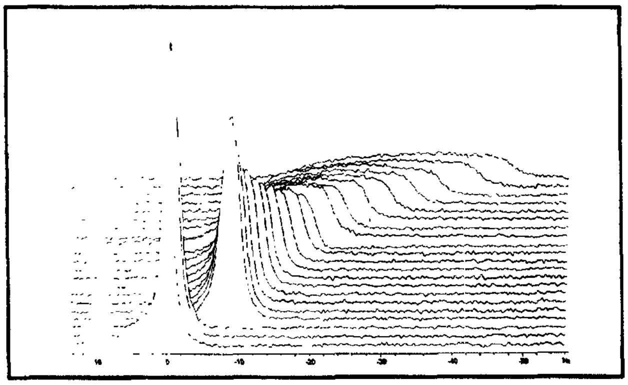

*Resonance shift and broadening during CE.*

### On-line Raman detection of microchip electrophoretic separations of neurotransmitters

*Karen A. Reshni, Robin M. Bright and Michael D. Morris, Department of Chemistry, University of Michigan, Ann Arbor, MI 48109-1055, USA*

We will describe Raman detection of electrophoretic separations in the microchip format. Use of the microchip improves both the electrophoretic and spectroscopic properties of a CE/Raman system. Coupling Raman spectroscopy to the microchip format is simple. The microchip, which is essentially a modified microscope glass slide, is held stationary on the microscope stage of an unmodified Raman microprobe. The flat surface of the microchip and its thin coverslip are the system for which microscope optics are designed. Focusing the excitation laser into this system with a high NA objective allows more efficient collection of Raman spectra than is possible with discrete capillaries.

Normal Raman spectroscopy allows both quantitative and qualitative analysis of analytes. It is hindered by a high concentration detection limit. We employ both preconcentration and spectroscopic enhancement techniques to decrease detection limits. Field-amplified injections, isotachophoresis (ITP) and isoelectric focusing (IEF) are the main techniques used to preconcentrate analytes. Concentration factors of 102–104 are obtainable. Surface-enhanced Raman spectroscopy (SERS) is also used to decreases detection limits through increased Raman scattering intensity. SERS spectra are $10^3 \times - 10^6 \times$ stronger than normal Raman spectra. Both preconcentration and SERS are necessary to enable measurements of neurotransmitters, e.g. biogenic amines, initially present in the biologically significant range of $10^{-6}$ to $10^{-8}$ M. They must be brought into the spectroscopic measurement range of $10^{-2}$ to $10^{-4}$ M. We will describe progress toward ultra-sensitive analysis using microfabricated SERS substrates incorporated directly into a microchip. SERS spectra are obtainable at lower concentration, but require a more complex microfabricated system.

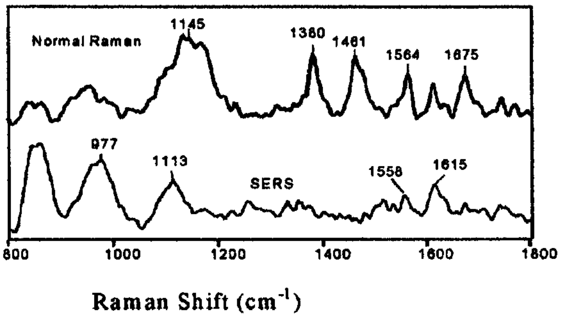

### A commercial fully automated 96-capillary array DNA sequencer

*Qingbo Li, Thomas Kane, Changsheng Liu, Harry Zhao, Robert Fields and John Kernan, SpectruMedix, 2124 Old Gatesburg Rd, State College, PA 16803, USA*

A commercial high-throughput DNA sequencer has been developed based on a robust multiplexed 96-capillary electrophoresis system and a high-performance replaceable gel matrix. The instrument is fully automated with the operation (e.g. sample introduction, DNA separation, instrument reconditioning, data processing) carried out and controlled by the instrument computer. The detector system employs on-column laser-induced fluorescence detection. It involves no moving parts. An air-cooled argon ion laser is efficiently coupled with the optics to excite fluorescence from all 96 capillaries. The instrument is easily movable. In the sample introduction system, a carousel assembly allows the automatic processing of seven 96-well sample trays without human intervention.

The high throughput arises primarily from four advantages: (i) the instrument design that allows complete automatic operation; (ii) the use of 96 discrete capillaries, with which 96 separate DNA samples can be analysed simultaneously; (iii) the use of a high-speed CCD camera that allows simultaneous monitoring of fast separation in all 96 capillaries; and (iv) the use of a dilute replaceable gel matrix that minimizes the time required for gel filling and capillary reconditioning. Including the time for sample introduction, separation and capillary reconditioning, the instrument is capable of one complete run within 2 h. Sequencing of at least 500 DNA bases per capillary is routinely achieved. The sequencing throughput of half a million bases per day is readily achievable using this 96-capillary instrument.

### On-the-fly fluorescence lifetime detection for DNA restriction fragment analysis by capillary electrophoresis

*Sara L. McIntosh, Brian K. Nunnally and Linda B. McGown, Department of Chemistry, P.M. Gross Chemical Laboratory, Box 90348, Duke University, Durham, NC 27708, USA*

The use of on-the-fly fluorescence lifetime detection for DNA restriction fragment analysis by capillary electrophoresis is described. Capillary electrophoresis is interfaced with a frequency domain, fluorescence lifetime fluorometer, which provides intensity and lifetime detection in no more time than is required for intensity alone.

We have investigated novel intercalating dyes as possible candidates for our ultimate goal of multiplex detection in fragment analysis, in which both a restriction fragment digest and a DNA ladder (size standard) could be run simultaneously in the same column and the fragments of several unknown digests distinguished from each other and from the ladder on the basis of fluorescence lifetime. These dyes are excited by the 488 nm or 514 nm line of an argon ion laser and have fluorescence lifetimes in the range of 0.5–3 ns. We observe baseline resolution for various dye-labelled 100-base pair DNA ladders and pBR322 DNA-BstNI digests, while near baseline resolution is achieved for the dye-labelled pBR322 DNA-MspI digests.

Our results indicate that, while excellent resolution of intercalating dye-labelled DNA fragments is achieved when only one digest is run at a time, multiplex detection may be difficult due to dissociation of the dyes from the DNA during the run. Fluorescence emission studies of numerous pairs of dyes have shown that there is indeed displacement of the dye with less affinity for the DNA when a higher affinity dye is added. These results have led us to investigate the possibility of using a covalently labelled DNA ladder (size standard), while still using an intercalating dye to label the DNA digest. This would allow simultaneous analysis of a digest and a DNA ladder during a single electrophoretic run and retain the ease of labelling of the DNA digest by using an intercalating dye.

### Extracting features from artificial neural networks models with sensitivity analysis

*Chuanhao Wan, Peter De B. Harrington, Kent J. Voorhees[1], Franco Basile[1] and Alan D. Hendricker, Ohio University Center for Intelligent Chemical Instrumentation, Department of Chemistry and Biochemistry, Clippinger Laboratories, Ohio University, Athens, OH 45701-2979, USA; [1]Chemistry and Geochemistry Department, Colorado School of Mines, Golden, CO 80401, USA*

Artificial neural networks (ANNs) have been widely used for classification in various chemical applications. Typically, the neural networks are full-spectrum methods in that the entire spectrum is used as an input object. A disadvantage of ANNs is that they function as black boxes and do not provide any diagnostic information regarding the characteristic features that are relevant for the classification. Furthermore, this information may contain useful information for establishing causal relationships and understanding the chemical information in the data. A procedure referred to as sensitivity analysis can be used to extract this useful information. A sensitivity spectrum is obtained by calculating the partial derivative of each input variable with respect to the change in output of the neural network. Our method is novel in that the ANN model is non-linear and the sensitivities are calculated about the mean spectrum for key classes. This method will be evaluated with neural networks applied to synthetic data and pyrolysis mass spectral data (obtained from EI mode) of bacteria. A radial basis function neural network (RBFN) has been trained to successfully classify the bacteria categories. The characteristic features used by the ANN in the full mass spectral data have been compared with those extracted from principal component compressed data for each class of bacteria.

### Rapid optimization and minimal complexity in neural network multivariate calibration of chlorinated hydrocarbons using Raman spectroscopy

*William J. Egan, Stephen L. Morgan and S. Michael Angel, Department of Chemistry & Biochemistry, The University of South Carolina, Columbia, SC 29208, USA*

Research into the theoretical and practical aspects of the use of neural networks (NNs) for non-linear calibration and pattern recognition in analytical chemistry has undergone a rapid increase in the last decade. Given the wide-ranging applicability and uses of neural networks, improvements to the NN modelling process are highly desirable. The number of adjustable parameters, or 'weights' of the network can be quite large, requiring a lengthy time to optimize the model. For example, a three-layer NN with 554 input variables, from spectral absorbance, and seven neurons would have 3893 weights and likely take 50 or more min to train via the standard backpropagation algorithm on a Pentium® class PC, even with a simple line search to adjust the learning rate. Further, once a NN is trained to produce satisfactory results, the interpretation of the weight patterns and connections is quite problematic. The intertwined, distributed nature of the weights hinders and often prevents the assignment of any particular weight to any particular input, making determination of physical or chemical properties difficult.

We have developed improvements to the NN modelling process with the goals of enhancing the optimization process and reducing NN model complexity. The NN modelling process may be divided into five steps: (i) preprocessing the input data; (ii) determination of the functional form of the NN model; (iii) initialization of the weights; (iv) application of an optimization algorithm; and (v) complexity reduction/model robustness. Improvements to the optimization process not only speed computation, but also can enhance the quality of the result. Complex NN models require more intensive optimization procedures and are considerably more difficult to interpret. Performance of these new algorithms will be demonstrated by results from training neural networks to correctly quantitate composition of mixtures of chlorinated hydrocarbons based on their Raman spectra.

### A genetic algorithm for pattern recognition analysis of chromatographic and spectroscopic data

*Barry K. Lawne and Anthony Moores, Department of Chemistry, Clarkson University, Potsdam, NY 13699-5810, USA*

The development of a genetic algorithm (GA) for pattern recognition analysis of chromatographic and spectroscopic data is reported. The GA selects features that optimize the separation of the classes in a plot of the two largest principal components (PCs) of the data, Because the largest PCs capture the bulk of the variance in the data, the peaks chosen by the GA convey information primarily about differences between the classes in the data set. Hence, the principal component analysis routine embedded in the fitness function of the GA acts as an information filter, significantly reducing the size of the search space, as it restricts the search to feature sets whose PC plots show clustering on the basis of class. (If a plot of the two largest principal components for a set of features yields well separated classes, one can only conclude that the bulk of the variance encoded by these features contains discriminatory information about the classes in the data set. Such features usually produce a good classifier.) In addition, the algorithm can focus on those classes and or samples that are difficult to classify as it trains using a form of boosting. Samples that consistently classify correctly are not as heavily weighted in the analysis as samples that are difficult to classify. Over time, the algorithm learns its optimal parameters in a manner similar to a neural network. The proposed algorithm integrates aspects of artificial intelligence and evolutionary computations to yield a 'smart' one-pass procedure for pattern recognition. The efficacy and efficiency of the pattern recognition GA is demonstrated using several chromatographic, Raman and near-IR data sets.

### Chemometric determination of column efficiency and unretained component dead time in multiple peak partition chromatography

*U. L. Peri-Okonny, Augustine Onwubuya, Wei Zeng and J. T. Maloy, Department of Chemistry, Seton Hall University, South Orange, NJ 07079, USA*

We have recently used finite difference simulations to demonstrate that the modified efficiency equation

$$\mathcal{N}' = t_R(t_R - t_0)/\sigma^2 = (\mathcal{N}/t_R)\cdot(t_R - t_0)$$

provides better peak to peak reproducibility of the plate count than the conventional efficiency estimate, $\mathcal{N}$, when a partition mechanism is operative in liquid chromatography.

This equation may be rearranged to the form $t_R = \mathcal{N}'(t_R/\mathcal{N}) + t_0$, where both variables $y = t_R$ and $x = t_R/\mathcal{N}$ may be determined for each digitized peak in a multiple peak chromatogram using statistical moment analysis; the constant factors $\mathcal{N}'$ (slope) and $t_0$ (intercept) may then be determined for the entire run using linear regression. This computational method not only allows a singular column efficiency to be reported; it also provides an unambiguous measure of the retention time of the unretained component for the purpose of capacity factor determinations. The standard errors of $\mathcal{N}'$ and $t_0$ may also determined by regression.

This paper addresses the computational issues associated with this treatment of the chromatographic data. As an indicator of the validity of the method, diagnostic computational criteria are presented that allow one to determine whether the partition mechanism is operative for a given chromatogram. In effect, each experimental peak is compared with the theoretical on-column bandshape that has been derived for partition chromatography

$$f(t, t_0, t_R, \mathcal{N}_0) = t_R/[2\pi\mathcal{N}_0(t_R - t_0)t]^{1/2} \times \exp\{-\mathcal{N}_0(t - t_R)^2/[2t(t_R - t_0)]\}$$

where $\mathcal{N}_0$ is the defined number of theoretical plates. Theoretical bandshapes obtained from this expression conform exactly to the equation of linear regression cited above. Thus, the partition mechanism may be considered to be operative when a set of experimental peaks conforms to this bandshape.

One such experimental system—the three-component aqueous buffer–methanol system of the pharmaceuticals ofloxacin (enantiomers) and ciprofloxacin (internal standard) on an Inertsil ODS column—is shown to meet these partition chromatography criteria and to yield remarkably constant values for $N'$ and $t_0$ when its digitized peaks are subjected to the prescribed numerical analysis.

### Rapid quantitative mixture analysis using a chemical sensor and chemometric regression

*C. Kai Meng and Philip L. Wylie, Hewlett-Packard, 2850 Centerville Road, Wilmington, DE 19808-1610, USA*

A new chemical sensor based on the HP mass selective detector (MSD) has been designed for rapid QA/QC or R&D analysis. The device consists of a headspace autosampler coupled directly to a quadruple mass spectrometer (MS) with chemometrics software for data analysis (pattern recognition) and model building. The volatiles from the headspace of the sample vials are sent to the mass spectrometer without any separation. Therefore, each analysis can be performed in 3 min or less.

Using the partial least square (PLS) algorithm in the chemometrics software, the chemical sensor can be used to quantitate a continuously changing property of a given sample type. For example, it has been used to determine the amount of adulterant oil in more expensive olive oils and to predict the peroxide value of butter samples, which is an indication of their age.

The chemical sensor takes advantage of the wide dynamic range, high selectivity and high sensitivity afforded by the MS to better quantify bulk components in mixtures than traditional electronic noses can.

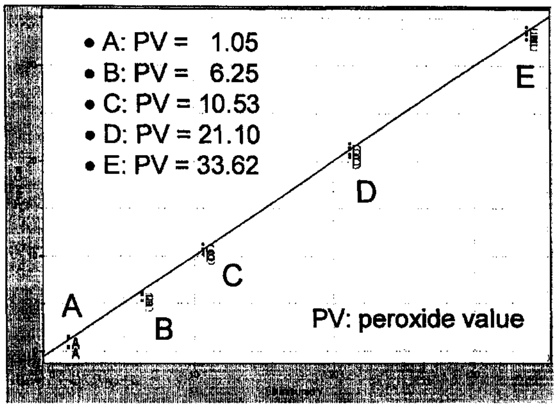

*Partial Least Square (PLS) algorithm was used to predict the peroxide values of butter samples* analysed by the chemical sensor.*

** Butter samples provied by Vince Shiers at Leatherhead Food Research Association in the UK.*

### Quantitating poorly resolved chromatographic peaks on single-channel detectors

*Christoph Klawun, Applied Automation, P.O. Box 9999, Bartlesville, OK 74005, USA*

For many years, chromatographic peak detection and quantification from single-channel detectors has been carried out by numerically determining the area of well-resolved peaks. Over time, this method has been shown to be very reliable and reproducible, amenable to any peak shape or baseline slope. Many algorithms have been invented to handle even the oddest separation.

In light of such a success of proven algorithms, why should it be necessary to tamper with working concepts? Occasionally, baseline separation of two or more peaks cannot be achieved by changing chromatographic conditions, columns, introducing multidimensional separation, or all of the above. Even more often, the cost of using the entire chromatographer's arsenal will be too high for fully separating the components of a mixture. However, a peak resolution of $<1.0$ may be seen as too unreliable, particularly when the concentration of the components have different orders of magnitude.

Several approaches have been suggested to quantify unresolved chromatographic peaks with $R < 1.0$ on single-channel detectors, e.g. FIDs, TCDS or RIDs for liquid chromatography. More approaches exist, e.g. fitting exponentially modified Gaussian curves to peak clusters. This paper will examine several of these approaches in practical applications, including potential savings in method development time, separation length and hardware complexity.

### Cascade correlation neural networks using Fourier and wavelet compressed data

*Chunsheng Cai and Peter de B. Harrington, Center for Intelligent Chemical Instrumentation, Department of Chemistry and Biochemistry, Clippinger Laboratories, Ohio University, Athens, OH 45701-2979, USA*

Compression of analytical data is re-emerging as an important research object, as chemical sensors are reduced size, lowered cost and increased in use. The sensor data may have to be stored on miniaturized devices, which may have limited storage capacity. Wavelet transform (WT) provides a powerful means to compress a large amount of data. The WT is a linear operation, which means the transformed coefficients can be directly input to other linear operations to perform multivariate analysis, eliminating the need to restore the data back to the original domain.

Temperature-constrained cascade correlation neural networks (TCCCNs) are powerful chemometric methods for pattern recognition and quantition. However, the training usually is very time consuming. Numerous methods have been proposed to speed the training step. Besides, care must be taken to reduce overfitting. Fast training using compressed data is presented. The WT-TCCCNs have two advantages: faster training and better results. The neural networks are trained using the WT compressed data. For chemical signals, the compression is very efficient and very little information is lost by compression, the data size is reduced significantly and the high-frequency noise is removed. As a result, the training speeds up and the possible overfitting to noise is avoided. Data obtained from ion mobility spectrometry (IMS) were used to evaluate the WT-TCCN method. The WT-TCCCN works very well for IMS data because IMS spectra usually have relatively uniform peak widths so that a range of wavelet types may be suitable.

### Optimization and validation steps for principal component regression (PCR)

*Frank Vogt, Klemens Rebstock and Maurus Tacke, Fraunhofer Institute of Physical Measurement Techniques, Heidenhofstr. 8, 79110 Freiburg, Germany*

Principal component regression is often used to evaluate measurement results quantitatively, especially optical spectra. In practice, the validity of the calibration set is tested only once, i.e. following the calibration process, but not during routine measurement or monitoring tasks. If system parameters change with time, e.g. due to baseline drifts, this may cause major errors. One focus of this paper is detection and correction of such time- and wavelength-dependent baseline variations. Normally, the calibration spectra and concentrations are mean centred in order to subtract an offset. If this offset changes in time, an uncorrected offset remains and the model built by the calibration is not validated any longer. We report on an inclusion of such drifts into the set of principal components (PCs) lifting this problem. This is performed by introducing a constant 'pseudo' PC, one linear in the wavelength, one quadratic term, etc. until the drift in a spectrum is modelled. From our experience, this procedure is superior to fitting a polynom to the mean-centred measurement spectrum. The information content of the PCs obtained by a singular value decomposition is tested in order to balance bias and introduce noise. Omitting a PC with low information increases the bias of the result, whereas included PCs add to the uncertainty of the result due to noise in the spectra. This procedure is performed for each measurement spectrum in order to minimize the uncertainty of the results and to obtain an individual statement of error. After calculation of the loadings of a measurement spectrum by least-squares fitting it to the PCs, the variance of the spectrum is calculated in order to determine the quality of the fit. Then conventional error propagation, involving this variance of the spectrum, is applied to estimate the uncertainty of the individual loadings. If this uncertainty for a specific PC outweighs the absolute contribution, this PC is omitted for evaluation resulting in a bias. If not, the PC is included and the uncertainty of the loading is transformed to an uncertainty of concentration. The bias is added to the sum uncertainty due to noise by root mean square.

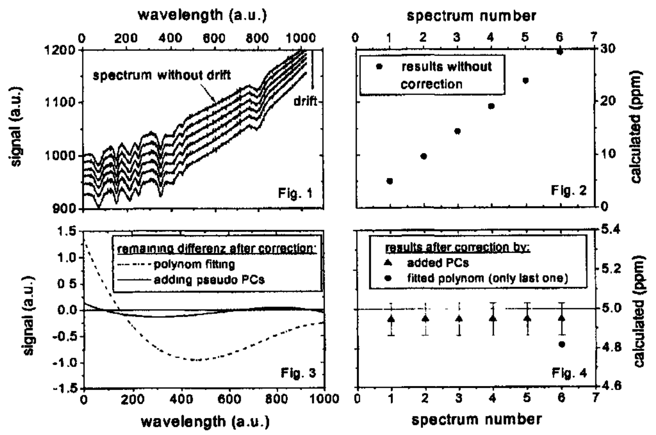

### A simple and rapid digestion procedure for determination of trace level arsenic in food by ICP-MS

*Reshan Fernando, Olujide Akinbo, John Soles, Amal Essader and Edo Pellizzari, Research Triangle Institute, 3040, Cornwallis Road, RTP, NC 27709, USA*

A simple and rapid digestion method has been developed for the determination of arsenic in food samples at trace levels by inductively coupled plasma mass spectrometry. The method involves digestion of 2–4 g of homogenized food using a two-stage, open-vessel, atmospheric pressure, controlled temperature microwave digestion method in the presence of 5 ml of nitric acid. A number of different food samples containing varying amounts of fat, fibre, carbohydrate and protein have been digested and analysed to evaluate the performance of the method. Several certified reference materials containing both organic and inorganic forms of arsenic were also used to evaluate the method performance. Digested samples were analysed directly for arsenic by ICP-MS. The instrument was calibrated using calibration standards from 0.050 to 10 ng As/ml prepared in 2% (v/v) $HNO_3$ to match the acid content in samples. Analysis results have indicated acceptable recoveries of arsenic from both certified reference materials and spiked food samples. Effects of the sample matrix, especially the chloride content in food, on the arsenic signal at mass 75 were investigated and found to be minimal under the conditions employed in the sample analysis. Under the analysis conditions employed, accurate results were obtained for sample chloride concentrations up to 4000 μg $Cl^-$/ml. Method performance evaluation and sample analysis results for arsenic in different food matrices will be presented in this paper.

### New automatic sample preparation approach to the GC and GC/MS ultra-trace analysis delivering faster results and increasing sample throughput of environmental laboratories

*Fausto Pigozzo, Paolo Magni[1], Fausto Munari[1], Albino Sironi[1] and Sorin Trestianu[1], CE Instruments, Austin, USA; [1]CE Instruments, Milan, Italy*

Conventional methods for the analysis of pollutants in water by capillary GC and GC/MS require extensive sample preparation procedures in order to meet the sensitivity requirements. Large-sample volume injection (LVI) of diluted solutions permits the elimination of preconcentration steps increasing the precision and accuracy of the analysis, simplifying and shortening the sample preparation procedures.

This paper describes a single-step sample preparation procedure consisting of a micro liquidliquid extraction performed directly into the autosampler vials, followed by automatic, large-sample volume injection into the capillary GC or GC/MS systems. The 'in vial' extraction is performed by mixing 1 ml of water sample with 1 ml of an appropriate organic solvent.

The cold on-column/desolvation precolumn LVI technique used for introducing the sample into the capillary GC or GC/MS systems permits the control of the injection and desolvation processes through a dedicated software. The software, described and illustrated in this paper, models these processes and automatically chooses the most appropriate operating conditions.

The advantages of this approach are illustrated by the results obtained from the analysis of traces of phenols, nitro and chlorophenols, organochloro and nitrogen and phosphorous containing pesticides, PAHs, PCBS, hydrocarbons and halocarbons in ground waters at ppb or ppt levels.

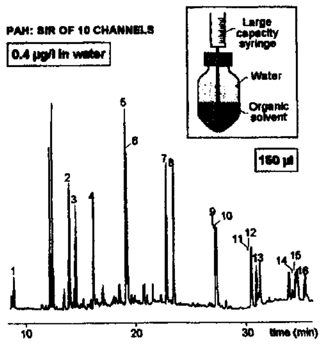

| Peaks | Components | RT (min) | RSD % | Area (counts) | RSD % | Recovery % |
|---|---|---|---|---|---|---|
| 1 | Naphtalene | 6.919 | 0.174 | 3572813 | 4.29 | 95 |
| 2 | Acenaphtylene | 11.425 | 0.018 | 3856321 | 1.68 | 99 |
| 3 | Acenaphtene | 11.954 | 0.041 | 3278637 | 0.73 | 98 |
| 4 | Fluorene | 13.472 | 0.038 | 2422061 | 1.27 | 98 |
| 5 | Phenanthrene | 16.156 | 0.031 | 3967819 | 1.82 | 99 |
| 6 | Anthracene | 16.306 | 0.030 | 3859096 | 0.71 | 98 |
| 7 | Fluoranthene | 19.578 | 0.027 | 3837532 | 0.33 | 98 |
| 8 | Pyrene | 20.160 | 0.023 | 4052069 | 0.73 | 98 |
| 9 | Benzo(a)anthracene | 23.720 | 0.018 | 3349898 | 1.49 | 95 |
| 10 | Chrysene | 23.801 | 0.021 | 3561044 | 2.34 | 98 |
| 11 | Benzo(b)fluoranthene | 26.602 | 0.019 | 3004859 | 3.73 | 98 |
| 12 | Benzo(k)fluoranthene | 26.673 | 0.018 | 3670001 | 1.09 | 94 |
| 13 | Benzo(a)pyrene | 27.349 | 0.016 | 3169913 | 1.70 | 97 |
| 14 | Indeno(1,2,3-cd)pyrene | 29.882 | 0.017 | 2472578 | 5.57 | 90 |
| 15 | Dibenzo(a,h)anthracene | 30.034 | 0.015 | 3611884 | 4.61 | 87 |
| 16 | Benzo(ghi)perylene | 30.372 | 0.016 | 3244002 | 4.26 | 91 |

*Large Sample Volume GC/MS analysis (selected Ion Recording on 10 channels, using CE Instruments TRACE GC 2000 coupled with Finnigan Voyager MS) of PAH-s in water (400 ppt each). In vial extraction with hexane and automatic injection of 150 µl (3 µl/s). UNCORET desolvation precolumn with solvent vapour exit, J & W DB5 MS capillary column, 15 m long, 0.25 mm ID, 0.25 µm film thickness. Oven temperature: 65 °C (3 min) to 290 °C (10 min) at 8 °C/min. He carrier gas: 80 kPa during desolvation and 1.5 ml/min in constant flow mode during the run.*

### Automatic injection of high-purity solvents with minimum carryover between samples

*Don E. Clay, Fausto Pigozzo, Rollen Anderson and Robert Wenske, ThermoQuest Corporation, 2215 Grand Avenue Parkway, Austin, TX 78728-3812, USA*

Petrochemical manufacturers are required to analyse a number of high-purity products, e.g. solvents or chemicals. Whether for quality control of shipments or process control in manufacture, these samples represent one of the most difficult problems in automatic sampling and analysis. In many cases, each of the chemical products is an impurity which must be controlled in the other products, usually at low ppm levels. This places stringent requirements on the amount of carryover between samples on an automatic sampler. For example, benzene, toluene, ethylbenzene and xylenes are chemicals that can all be analysed using the same gas chromatographic method on a single instrument. In actual practice, these samples are not often run automatically on a single instrument because of cross-contamination of the syringe used for injection. The tiny amount of residual sample remaining in a syringe after injection may be many times the amount of impurity in the other products. Even using multiple solvent and sample wash cycles does not satisfactorily reduce this carryover into the following sample. Current analytical methods must resort to manual injection with individual syringes dedicated for each chemical, or automatic instrumentation dedicated to a single product or chemical because of this carryover problem. This increases analytical costs enormously over a single-instrument method, and may frequently create a bottleneck to high productivity.

This paper describes an autosampler for gas chromatography that reduces carryover to acceptable levels. A combination of flexibility in autosampler operation and proper choice of syringe type and capacity are needed to solve this problem. Wash cycles with solvent and sample, syringe draw rate, and the number and speed of syringe bubble elimination cycles all have a significant impact on carryover from one pure solvent to another. Data are presented showing the magnitude of carryover in standard operation, and a comparison demonstrating reduced carryover with optimum autosampler and syringe parameters. This will allow operation of a single instrument with an autosampler to take the place of multiple dedicated instruments.

### Intelligent multivariate optimization for multi-stepwise gradient elution in HPLC

*Yukui Zhang, Ruijiang Lee, Longzhu Jin and Hanfa Zou, National Chromatographic R & A Center, Dalian Institute of Chemical Physics, Chinese Academy of Sciences, Dalian, 116011, P.R. China*

Though the optimization of high-performance liquid chromatography (HPLC) has received more and more interest, and many optimization methods have been developed, there still were not effective methods to solve the problem of separation of complicated sample optimization of separation condition in HPLC, especially the multivariate optimization for separation condition of gradient elution. In this work, a new method is developed and its related soft-package is designed to optimize multi-stepwise gradient and multivariate composition of the mobile phase in HPLC and RP-IPC. The optimization strategy is summarized as follows:

(1) Prediction basis. The gradient retention time and peak width of solutes under multi-stepwise gradient elution are predicted by equations obtained by a uniform design-iteratire optimization procedure.
(2) Dynamic grouping. Considering the length that the individual component has moved in the column at the optimized isocratic or gradient separation condition, its elute time and resolutions with adjacent peaks are predicted. Then, the components still in the column are dynamically grouped to obtain the group of components that should be separated at the next step.
(3) Step optimization. The mobile phase composition of a new gradient step is determined through an isocratic optimization for the group of components obtained by dynamic grouping. The time of the gradient step is determined not only by the elute time of the least separated pair of solutes in this gradient step, but also the resolution of the solutes following it and the delay time of the system.

(4) Global optimization. In step optimization, for every solute, position in column, time it will be eluted out of column, peak width and resolution with adjacent peaks are simulated instantly and point by point in the parameter space, and the value of optimization criterion is calculated. This ensures the global optimal composition of the mobile phase is obtained for every gradient stepwise.

The optimization strategy is confirmed to be an effective strategy by the separation of 18 amino acids derived with 2,4-dinitrofluorobenzene (DNFB), 21 anilines in HPLC, and 24 sulphonic acids in RP-IPC.

### Productivity enhancement tools for LC/MS: automated method development system for mobile phase selection

*Steven D. Cubbedge, Arthur J. Boyer and Robert Classon, Shimadzu Scientific Instruments, 7102 Riverwood Drive, Columbia, MD 21046, USA*

Many liquid chromatography laboratories now use mass detection as a routine analytical tool. In many cases the results include decreased analysis time, high throughput and better measurement integrity. However, this increased power and functionality often come with increased complexity and greater operator training. This is especially true in the LC/MS arena when chromatographers add a mass detector to their HPLC for the first time.

Often, LC methods do not translate directly to LC/MS. Detection by LC/MS relies on the ionic state of the compound in solution. Electrospray (ESI) methods generally require analytes to be charged in solution. Atmospheric pressure chemical ionization (APCI) is more efficient when the neutral species is present. The ionic state in solution is determined by the compound and mobile phase composition. These also influence the chromatographic retention time and resolution. Understanding these properties in advance requires detailed knowledge of the compound of interest, most importantly the pKa for each proton on the analyte. Many times this information is not available, and thus can be problematic.

Previously, solving these problems required extensive LC/MS experience. Because many chromatographers are interested in changing to MS detection, tools have been created to help them. To ease this transition, an automated system has been developed utilizing solvent selection valves, a fully programmable autosampler, LC/MS and a PC-based data system which automatically analyses a sample with a variety of mobile phases. The goal of this development is automated optimization of atmospheric pressure ionization LC/MS with minimal user intervention.

The experimental design and system will be described and results will be presented in this paper which demonstrate the effectiveness of the system in finding mobile phase compositions. This process promotes good ion yield and provides users with detailed reports documenting their work.

### Development of new automated mass spectrometry systems to accelerate drug discovery and development

*Joseph F. Anacleto, John Robson, Eva Duchoslav and Ron Bonner, Perkin-Elmer Sciex Instruments, 71 Four Valley Drive, Concord, Ontario, Canada L4K 4V8*

The advent of combinatorial chemistry and high-throughput organic synthesis has increased the number of potential lead compounds that may continue to further development. This has created an increasing demand on bioanalytical chemists to provide efficient methods for producing data that can be used for selecting and further developing suitable candidates. One approach for producing this data is the use of CACO-2 cell systems which are amenable to the high-throughput requirements stemming from the large numbers of new compounds that need to be tested in a short time. With this approach, the time-consuming step was the analysis of these large numbers of samples. The analytical challenges were: (i) how to assay samples for multiple compounds in one automated batch; and (ii) how to process the large data set in an automated fashion.

In this report, we describe an automated LC-MS/MS system that can be used for the unattended analysis and data report generation for a series of compounds. The system will automatically determine the optimum MS/MS conditions for a set of compounds. This includes selecting the orifice voltage, polarity, collision energy and most intense product ion for each compound. It will then automatically build experiments and batch files from the optimized conditions data. The acquired data can also be automatically processed and the results presented in various formats. By automating compound optimization and mass spectrometric method building (removing the need for time-consuming manual procedures), the LC-MS/MS system accelerates method development and greatly increases sample throughput. The simplicity and 'ease-of-use' of the software makes it ideal for non-expert users.

### Integrated LC/MS system software based on Windows NT for unified hardware control and data management

*Craig A. Walla, Andre Szczesniewski, Peter B. Grosshans and Ruidan Chen, Hitachi Instruments, 3100 North First St., San Jose, CA 95134, USA*

Hitachi has developed an integrated LC/MS data station for our LC/3DQ® ion trap mass spectrometer. This data station integrates a complete HPLC system including pump (isocratic, low- and high-pressure gradient modes), autosampler, single-channel detectors and column oven with the ion trap mass spectrometer and any of four API interfaces. The mass spectrometer supports many features including autotune, full scan with unit mass resolution, segmented scanning, tandem MS, multiple interleaved experiments and quantitation. Hitachi has designed this data station for ease of use by both chromatographers and mass spectrometrists.

The user interface is simple and intuitive so that non-MS specialists can operate the system on a daily basis. While being easy to use, this UI supports the needs of high-end users by allowing full control of the MS to help solve their most demanding applications.

The API interfaces (APCI, ESI, SSI, Semi-Micro APCI) are designed to be as simple and intuitive as possible. This applies not only to the hardware, but also to the software where pictures of the interface are used as the basis for the method setup screen.

This paper will show how this data station combines HPLC and MS to provide an integrated solution. The result is a simple to use software/instrument combination that delivers high-quality data.

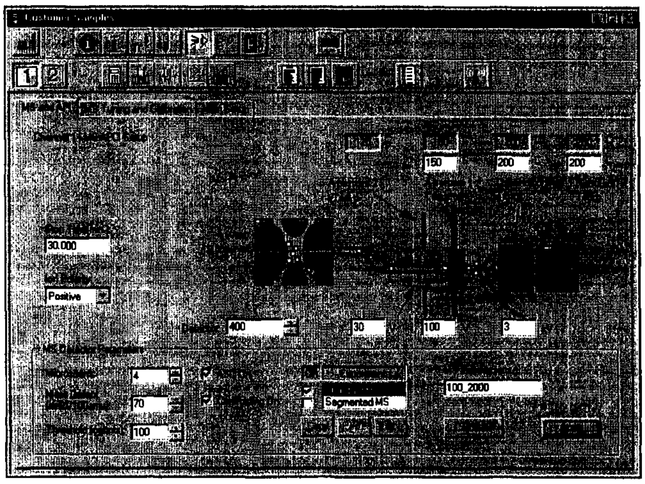

*MS and APCI setup screen.*

### NIR analysis of diesel fuel properties

*Zhao Lu and Brian Curtiss, Analytical Spectral Devices, Boulder, CO, USA*

The properties of diesel fuels determine its suitability for specific use. Most often the evaluation of fuel quality needs to be carried out in the lab and is time consuming. Thus, a rapid, simple, yet accurate measurement technique is desirable.

In this paper, we demonstrate that the near-infrared (NIR) spectroscopy technique meets all the above requirements. The portable NIR spectrometer we developed is lightweight and easy to couple with many types of fibre optic probes. With this instrument coupled with transmission probe, we acquired absorbance spectra of fuel samples with known properties, e.g. cloud point, pour point and flash point. Multivariate calibration models were developed to estimate each of the properties of interest. Both linear and non-linear models are discussed and evaluated by a separate validation data set. The results presented in this paper show that NIR is a simple and accurate method that provides information within a minute. In addition, the portability of the instrument makes the analysis much more flexible and can be performed either in the field or in the lab.

### Real time process optimization using a high-pressure fibre optic flow cell: destruction of spent dye baths using supercritical water oxidation

*Rajesh Paradkar, Matthew Williams, Michael Barr and Michael Drews, School of Textiles, Fiber and Polymer Science, Clemson University, Clemson, SC 29634-1307, USA*

Supercritical water oxidation (SCWO) is a promising technology that can be used for complete and efficient oxidation of hazardous waste streams at moderate temperature and high pressures, without the formation of harmful by-products. In order to investigate the use of SCWO as an approach to treating aqueous waste streams from textile wet processing, a small-scale SCWO reactor was built and tested using disperse dye bath and sulphonated lignin (black liquor) waste streams. As expected, extensive colour and lignin destruction, and significant lowering of both TOC and COD of the reactor feed streams was observed. Problems were encountered in attempting to model the process because the SCWO system is a semi-batch process, and significant mixing and dilution occurred in the reactor feed cell. As a result, the reactor feed concentration (as a function of time) was largely unknown.

In order to overcome this problem, we have designed and tested a high-pressure fibre optic flow cell and spectroscopic monitoring system (UV-Vis, 220–800 nm). This flow cell uses quartz windows, holds 300 atm at 127 °C and allows for continuous monitoring of the reactor influent and effluent. This in turn allows for real time control and optimization of flow rate (residence time) through the reactor, i.e. lower concentration feed can be processed at a higher flow rate (lower residence time) relative to higher concentration feed, while obtaining similar per cent destruction of the feed. Results obtained using the fibre optic flow cell will be presented in this paper along with a detailed description of the SCWO system and the high-pressure flow cell.

### Automation of standard methods—permanganate time

*Mike Collins, Ron Belchamber, Lorelli Hilliard, Tom Lynch[1], David Lightowlers[1] and Paul Greenwood[1], Process Analysis and Automation, Fernhill Road, Farnborough, Hampshire GU14 9RX, UK; [1]BP Chemicals, Saltend, Hull HU12 8DS, UK*

Many industrial analyses rely on standard tests. These were often developed many years ago as simple means, e.g. for the determination of product purity. They are usually based on ASTM or pharmacoepia methods, entrenched in product specifications and difficult to change.

We have been investigating the automation of these tests. The results have been illuminating. Using modern analytical equipment and automated procedures, we have found:

- test time can be greatly reduced;
- accuracy can be improved;
- operator error, due to the subjective nature of these tests, can be eliminated;
- testing can be moved from the laboratory to at-line making the method suitable for incorporation into process control;
- sample tracking can be incorporated.

The 'permanganate time' test is used to detect easily oxidizable impurities in solvents, e.g. acetic acid, acetic anhydride, ethanol, methanol and pyridine. The standard test involves adding permanganate solution, waiting for a specified period of time and a visual check whether the pink permanganate colour has been discharged.

We have automated this procedure using optical dip probes, a photodiode array spectrophotometer (Carl Zeiss) and virtual instrumentation software developed using LabVIEW (National Instruments). Instead of waiting the specified time for the colour change, we are able to predict the permanganate time by observing the initial rate of decay of the permanganate. The whole system has been incorporated into a rugged, at-line instrument.

### Vibrational spectroscopy software and databases: a vendors' perspective

*Michael P. Fuller, Nicolet Instrument Corporation, 5225 Verona Road, Madison, WI 53711, USA*

In the evolution of FT-IR instruments from primordial slime of the university physics labs to the marketing madness that surrounds a mature high-tech product, there have been many remarkable developments. While product spec sheets over the years have touted the spectrometers signal-to-noise ratio advances and the improved long-term stability characteristics, it could be argued that the greatest advances have been made not in the spectrometer hardware but in the attendant software. Most FT-IR manufacturers have probably spent nearly as many R&D dollars on software development over the years as they have on improved optical designs. Changes in computers, operating systems and development tools have allowed software to emerge from the depths of three-letter command and parameter mnemonics to the advanced graphical user interfaces we are used to and expect in today's products. The performance and reliability of commercial FT-IR spectrometers has advanced to such a high level that often it is really the software that distinguishes one brand from another. This paper will cover the advances in FT-IR software and databases over the last 30 years from the point of view of the instrument manufacturers.

### Chemometrics: a historical perspective and new opportunities with improved algorithms

*David M. Haaland, Sandia National Laboratories, MS0342, Albuquerque, NM 87185-0342, USA*

In the late 1970s and early 1980, multivariate methods for infrared spectroscopic analysis were being developed. In these early days, classical least squares (CLS) and inverse least squares (ILS) were applied for the first time to the quantitative analysis of mid-infrared spectral data. Each method had restrictions that limited its applicability. Later, the introduction of partial least squares and principal component regression methods offered many of the benefits of both CLS and ILS methods without their respective restrictions. This early history will be presented, and the relative benefits of each multivariate calibration method will be given. The historical discussion will be followed by the presentation of new developments in the application of CLS methods. We will show that the qualitative spectral interpretation of CLS has always been one of its strengths, but its precision has generally trailed that of PLS and PCR for liquid and solid samples. Recently, we have added new features to the CLS algorithm to make it capable of prediction precision for condensed phase samples that can be comparable to those obtained with PLS and PCR. These new capabilities also provide a means by which CLS can be used to improve calibration maintenance in the presence of spectrometer drift and can even be used to achieve multivariate calibration transfer.

The new CLS method, called prediction-augmented classical least squares (PACLS), allows the addition of spectral shapes of molecular components or spectral changes that were not included in the original calibration to be included in the prediction phase of the CLS analysis. It will be shown that this method negates the detrimental influence of these unmodelled spectral shapes on the predicted concentrations. The spectral shapes can be obtained from multiple spectra of a sample repeatedly monitored during calibration and/or prediction. In this case, the spectral shapes derived from the repeat sample spectra can be used in the PACLS algorithm to model spectrometer drift and to maintain a calibration on a drifting spectrometer. By calculating difference spectra for a subset of calibration samples measured on both primary and secondary spectrometers, we can generate the spectral shape differences between spectrometers as they are reflected in the sample spectra. Thus, calibration transfer can be accommodated with the PACLS method. Finally, the addition of the spectral shapes of components not included in the original calibration can be used to correct the detrimental effects of unmodelled components in the prediction of spectra obtained from unknown samples. Examples of each of these advantages will be presented using the spectra obtained from dilute aqueous solutions. All these new capabilities will increase the importance and use of CLS methods in the future.

### Spectroscopy and the Internet: a new model for research

*James H. Duckworth, Galactic Industries, 395 Main St., Salem, NH 03079, USA*

No single topic has dominated the press in recent history as the growth of the Internet. Although it was originally designed as a means for scientific collaboration, only with the recent commercialization of the Internet has there been significant interest in using its basic technologies to support spectroscopic research. There are a number of areas where the Internet has had an impact on spectroscopy ranging from bibliographic databases and on-line publications to complete research databases of spectroscopy and chemical property data. In addition, the Internet offers the potential for more collaborative research outside the laboratory. This paper will attempt to show some of the current uses of the Internet in spectroscopy research and point to some upcoming technologies.

### Optimization of the fabrication process for open tubular capillary separation columns prepared by the sol-gel method

*Shirley A. Rodriguez and Luis A. Colón, Department of Chemistry, State University of New York at Buffalo, Natural Science Complex, Buffalo, NY 14260-3000, USA*

An organic–inorganic hybrid composite was synthesized using sol-gel processing. A thin film of this composite was coated onto the inner wall surface of fused silica capillary columns, serving as the stationary phase for open tubular capillary electrochromatography (OT-CEC). Composites for reversed-phase OT-CEC have been fabricated by hydrolysis and condensation reactions of *n*-octyltriethoxysilane (C8-TEOS) and tetraethoxysilane (TEOS) at ambient conditions. Octadecyltriethoxysilane (C18-TEOS) and TEOS have also been used as precursors to fabricate materials suitable for reversed-phase OT-CEC. The alkyl-substituted precursor is incorporated in the glass formation process, leading to the glass composite with organic characteristics.

We have investigated the hydrolysis and initial condensation reactions of the C8-TEOS/TEOS and C18-TEOS/TEOS in order to understand the processing that leads to the final chromatographic performance. Using $^{29}Si$ NMR, the formation of the various silicon species and the degree of substitution on the silicon atom were followed. We observed that the rate of hydrolysis of each precursor is different when in a mixture (e.g. C8-TEOS/TEOS) than when the precursors are alone. We have also correlated the degree of condensation with chromatographic retention. The higher retentive characteristics were observed on columns coated at the time that the maximum degree of condensation was achieved.

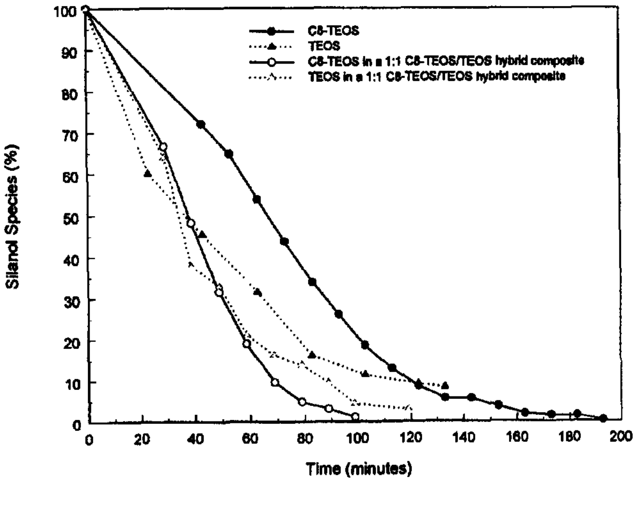

*Rate of hydrolysis of silane precursors.*

In this paper, we will discuss the details of the NMR experiments and their correlation to chromatographic retention. In addition, we will present the column preparation procedure for an optimized organic–inorganic hybrid material.

### Advancements in data management and software validation incorporated into new generation chromatography

*Kathy Wirt and Linda Jackson, Dionex Corporation, 1228 Titan Way, Sunnyvale, CA 94086, USA*

Modern chromatography laboratories require sophisticated software systems for data management and comprehensive documentation for regulatory compliance. To address these needs, a new chromatography data system built upon an advanced client-server architecture has been developed. This software features full network capabilities and supports remote maintenance via a modem. An integrated SQL database is incorporated for flexible sample management. Access to the data is simplified through the use of a browser modelled after the Windows 95™ Explorer. Dialogue boxes are used to define queries that are automatically translated to SQL. Extensive audit trail and validation features are supported, including multi-level password access and detailed event-logging.

### Trends and advances in chromatography data handling

*Peter W. Yendle, LabSystems R&D, 1 St George's Court, Hanover Business Park, Altrincham, Cheshire WA145TP, UK*

This paper presents LabSystems' view on a number of major trends in chromatography data handling. Specifically, the following issues are discussed.

(1) End-user productivity. There is an increasing demand to reduce the time and cost of analysis. Making the analyst more productive is therefore the key goal for any automation system.
(2) User-driven development. Involving users closely in system development is essential to meet the goal of increased productivity. System providers therefore need to adopt a development methodology which supports a joint application development (JAD) approach.
(3) Systems integration. To increase productivity, a chromatography data handling system needs to integrate with other systems in use in the lab. This includes integration with desktop tools, e.g. spreadsheets, word processors, etc. It also requires integration with the corporate computing environment, e.g. with laboratory information management systems (LIMS).
(4) System administration. To maximize the time available for analysis, the need for system administration should be minimized. This can be achieved by a combination of system design and the adoption of standards which facilitate administration.
(5) Instrument automation. Many labs utilize a wide range of chromatographic instruments. To increase productivity, a chromatography system needs to be able to interface to, and provide control for, the full range of these instruments.

### The open lab approach for the chromatographic and MS laboratory

*Franco Spoldi, ThermoQuest Corporation, 355 River Oaks Parkway, San Jose, CA 95134, USA*

The modern analytical lab can be described in a variety of ways. One approach focuses on different types of lab structure: single user in a laboratory, multiple users in a laboratory, and multiple users in a lab and with office access to data. An ideal software package should address these distinct requirements.

*Defining the ideal software package*

ThermoQuest resolved the multiple challenges of these requirements with the implementation of:

- a common platform operating system: Windows NT;
- two basic application software packages: one for chromatography, ChromQuest™; and one for mass spectrometry, Xcalibur;
- a uniform software philosophy: 'the open lab'.

The company chose two different applications packages because the needs for mass spectrometry data manipulation differ from chromatography. The 'open lab' concept transcended the individual laboratory scenarios. It was the unifying force, the optimal fit for the broad range of chromatographic laboratories.

*The open lab*

The open lab concept demands that software always provide:

- multiple technology functions;
- multiple vendors' instrument control;
- complete set of tools for all the applications in chromatography and mass spectrometry;
- complete scalability from single user to multiple users in multiple locations.

*Multiple technology functions*

ChromQuest software provides the system set-up of any combination of LC or GC systems achieved simply from one system configuration window. Users can monitor and control one system in a technique while another system of a comparable technique is running, Xcalibur software is the unifying data system for ion trap and single- and triple-quadrupole mass spectrometers. It also extends to control the complete range of ThermoQuest LC and GC instruments.

*Multiple vendors' instrument control*

ChromQuest collects data from PE Nelson's A/D interfaces and controls Hewlett-Packard GC systems. Xcalibur features a VIP program (virtual instrument partner) that embraces third-party instrument vendors. It empowers them to write an Xcalibur 'virtual instrument' for their device. That third-party device then becomes immediately usable in any appropriate Xcalibur mass spectrometer configuration.

*Complete set of tools for all the applications in chromatography and mass spectrometry*

ChromQuest features a spectral analysis package and easy integration with Windows applications. Xcalibur adds several applications to its base package, including $LC_{QUAN}$, BioWorks, Discovery (combinatorial chemistry) Freeware, Mass Frontier (high chem.) for spectral analysis. It also features layered applications, open access, ToxLab. The OCP program (object control partner) enables third-party hardware or software vendors to link to the MS control and data generated in Xcalibur.

*Complete scalability from single user to multiple users in multiple locations*

ChromQuest and Xcalibur are completely scalable from single-station to enterprise-wide software. Both software systems seamlessly integrate the scalability and security features inherent in Windows NT.

### Implementation of electronic nose and sensor array systems in an industrial environment

*John Poling, Quitterie Lucas and Frederic Zenhausern, Alpha M.O.S. America, 102 Towne Centre Drive, Hillsborough, NJ 08876, USA*

Manufacturers of products have a simple objective, deliver product that is acceptable to the customer and at the highest production yield. The impact of not meeting this goal can have a profound impact on the profitability of a company and the relationship they have with their customers. The consistency of production is at the mercy of several variables, e.g. the consistency of raw materials, manufacturing variations, and the unpredictable change in customer perception.

Over the past years, the use of sensor array technology or 'electronic nose' systems has been presented as a method of rapidly measuring a sample and objectively comparing that sample to a qualitative or quantitative model. The access to information prompted the manufacturing teams to take action and improve manufacturing efficiency.

The analysis provides the operator with the confidence to accept or reject raw materials, approve a production batch to the next stage, or release a finished product to the intended customer. The ability to make these decisions quickly and reliably saves time, reduces waste, and ensures customer satisfaction.

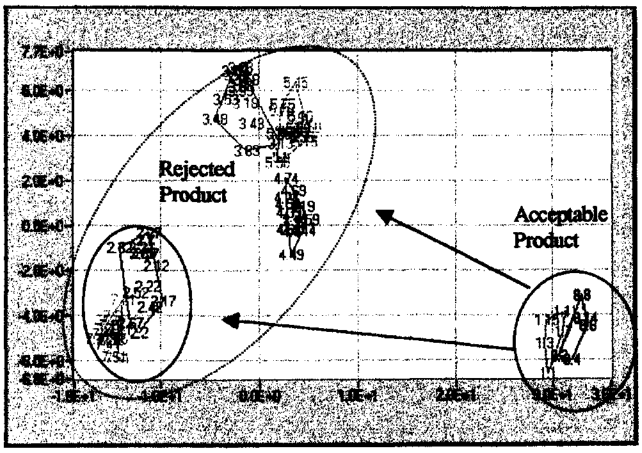

Turn-key systems which provide rapid, simple and reliable analysis with little or no sample preparation and rapid answers are needed in the process area to overcome analytical bottlenecks and to support the lab in providing information in a timely fashion. This paper will present the steps needed to take 'electronic nose' technology from the lab to the industrial process. Examples of process and industrial applications will be cited including the milestones and considerations to implementing sensor array technology in a process environment. A direct comparison of this shop floor technique to the cost of analysis by taste panels and analytical techniques, e.g. GC and GC/MS will be provided in this paper.

### Chemical sensors for electronic noses

*Joseph R. Stetter and Wolfgang Göpel*[1], *Illinois Institute of Technology, BCPS Dept., Chicago, IL 60616, USA;* [1]*Universitat Tubingen, Auf der Morgenstelle 8, D-72076 Tubingen, Germany*

Arrays of sensors can be used to approach analytical problems that single sensors cannot address. There are many different principles of operation for chemical sensors including acoustic, calorimetric, electrochemical, optical and chemiresistor. The performance of each sensor type produces various sensitivity, selectivity, response time and stability. The sensor must be a good performer for the sensor array to be a good performer, i.e. not all sensor arrays or E-noses are created equal.

Interpretations of pattern differences as related to an odour or particular endpoint can be a source of error. Examples include the identification of different wines based primarily upon their alcohol signals or identification of coffee odour based upon the coffee's relative humidity signal. The rules for qualitative analysis by pattern recognition for chemical sensor arrays require that: (i) a combination of chemicals that make each sample distinct exists; (ii) this chemical combination and the desired endpoint (e.g. odour, hazard, quality) are related statistically; (iii) the answer can be adequately represented by a set of responses from the particular sensor array used; (iv) a particular relationship can be found using the chosen pattern recognition algorithm (e.g. PCA, PLA, ANNs); and (v) the relationship remains valid and can be extrapolated to additional situations and unknowns.

It is obvious that the type of sensor employed can have a profound effect on the validity of the qualitative analysis performed by the E-nose. If more than one sensor type is included in the array, we have a heterogeneous chemical sensor array and a better opportunity to obtain excellent performance in a wider variety of situations. Using the MOSES II electronic nose (Lennartz Electronic, Tubingen, Germany, w.lennartz-electronic.de), several different analytical methods have been investigated. For some mixtures it is better to have a heterogeneous sensor array. For other analytical problems, a single array of either MOS, QCM or electrochemical sensors performs with better reliability. In any case, it is better to have the ability to sample in different ways, expose different sensor types to the sample, and utilize different pattern recognition schemes when developing a new method or validating an old one. Once the method has been optimized and thoroughly validated, a smaller, more focused sensor array and a simpler E-nose can be configured. MOSES II allows significant flexibility in the development of qualitative E-nose methods for food, industrial and biomedical applications.

### Chemical sensor arrays in process monitoring

*Howard Paul, Ley Hathcock and Mark Byfield, EEV Chemical Sensor Systems, 4 Westchester Plaza, Elmsford, NY 10523-1482, USA*

Instruments using sensor array technology, or so-called 'electronic noses', have been commercially available for several years. They have achieved various degrees of success in a wide range of applications, and most often take the form of laboratory-based instruments. The laboratory environment allows for careful control of external factors which may impact results, including ambient condition variations, and variations in sample or sample preparation. Outside of the laboratory, e.g. in a process-monitoring situation, these factors become much more important, and if not dealt with adequately, can overwhelm the capabilities of the technology.

This paper describes steps taken to make sensor array technology suitable for outside-of-the-laboratory applications. Using a modular approach, systems are designed which include factory-hardened sensor heads and sampling probes, industry standard interfaces and data handling platforms. These systems provide the ability to monitor odour on- or at-line in a manner not dissimilar to the current use of temperature, pressure, flow and pH sensors. The sampling hardware is generally minimally invasive, and resembles other common process-monitoring equipment. Interfaces and data-handling devices are industry standard, allowing easy incorporation into existing process-monitoring systems.

The fundamentals of sensor array technology have been re-evaluated with respect to the requirements of outside-the-lab applications. Critical issues, e.g. sensor drift, training and calibration are dealt with in an effective manner, which is suitable for the process-monitoring setting. Users of laboratory-based electronic nose instruments often require the flexibility of statistical and neural analysis packages. To achieve optimal results with these instruments, significant end-user data handling is necessary, and at the very least the data analysis itself can be confusing, if not daunting. This is sometimes at the expense of rapid results and high sample throughput. By automating data handling on processor platforms suitable for factory-type environments, real-time or near real-time results can be obtained and presented in classical QA/QC charting and output formats right alongside data from other process monitoring and control measurements. Alarm levels can be set and updated at user interface terminals on the factory floor.

### A powerful electronic nose by upgrading your (GC-LC)/mass spectrometer

*Heinz Roberg and Herve Boularot, ANALYT GMBH & Co KG, PF 1321, D-79379 Muellheim, country?*

A mass spectrometer combined with an appropriate statistical software becomes a powerful electronic nose. The system is reliable with fast response. Using up to 800 masses, i.e. 800 specific sensors, enables to handle any kind of samples: solids, liquids, vapours. It avoids the current problems of conventional sensor arrays (MOS, CP, QMB, SAW, etc.), e.g. lifetime of sensors, long-term stability, influence of humidity and temperature, and calibration problems.

MSstat™ is an easy to handle statistical (chemometric) software dedicated to operate beside your mass spec.: GC/MS, LC/MS or MS. It does not interfere with the hardware/software of the mass spec and does not require any hardware modification. The mass spec data files are copied into MSstat™. MSstat™ operates in the background of the PC performing automatic sample recognition. It upgrades a standard mass spectrometer (MS, GC-MS, LC-MS) to a powerful universal quality control instrument, suitable for chemical industry, food/beverage, packaging, inks, tobacco, diseases, pharmaceuticals, cosmetics, etc.

Calibration (training) is performed with real samples. These samples can be classified with respect to human criteria, e.g. good, bad, borderline, spicy, tasteless, etc. MSstat™ enables to find the set of masses which allow the enhancement of discrimination between these groups. Unknown samples can be classified with respect to these criteria.

Using a mass spectrometer combined with MSstat™ enables fast and reliable recognition of unknown samples. Analysis time can be drastically reduced (e.g. a GC/MS analysis with temperature program: analysis time = 74 min). The same analysis performed isothermally at high temperature with MSstat™: analysis time reduced to 14 min. At any time, the GC/MS can be used for standard analysis operation. The 'relevant masses' in terms of discrimination between groups provide useful chemical information. They indicate which masses, i.e. chemical components, are responsible for the differences. This means a direct link between qualitative results obtained with human criteria and results from analytical instruments. Such information cannot be obtained with standard electronic noses.

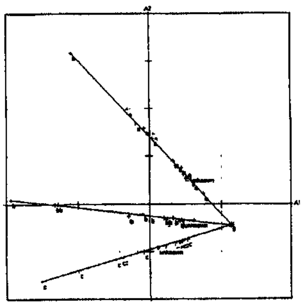

3 Flavours. 2D-Geometric Display

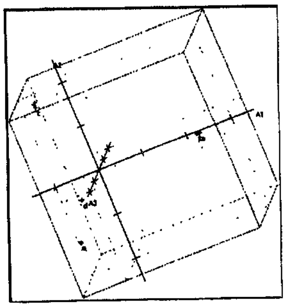

Vinegars. 3D - Display

### Application of pattern recognition and chemometrics to sensory array system

*H. Amine, S. Labreche and T. T. Tan, Alpha MOS S.A, 20, Avenue Didier Daurat, 31400 Toulouse, France*

The gas sensor array system has been used and validated in different fields, e.g. food and cosmetics. The aim of this paper is to define the different problems to be solved in the quality control of odours. These different problems can be divided into four categories.

(1) Good–bad: the objective is to define areas of good and to teach it to the system.
(2) Discrimination: this occurs when one wants to assess the difference between different types or origins of a given product.
(3) Quantification: the goal is to calculate the concentration of a given impurity present in the measured product.
(4) Sensory panel score correlation: after sensory panel training, the system is calibrated to predict the score of an unknown product.

For each of these categories, the methodology is obtained from the feasibility study of the validation phase. Once the methodology is defined, appropriate chemometrics and pattern recognition techniques are given. Different methods are studied, e.g. SIMCA, DFA, PDF for good–bad and discrimination application; PLS, MLR, PCR for quantification and correlation with sensory panel score. Outliers detection and validation methods are also discussed.

Finally we will show some results on each of these categories and discuss how to make the methodologies automatic for easy-to-use systems, e.g. a dedicated instrument.

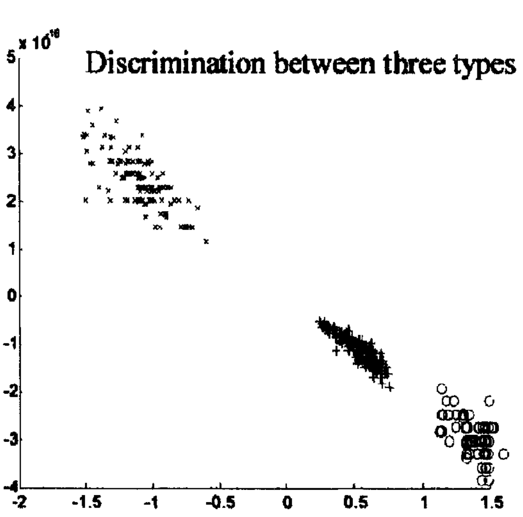

*Discrimination*

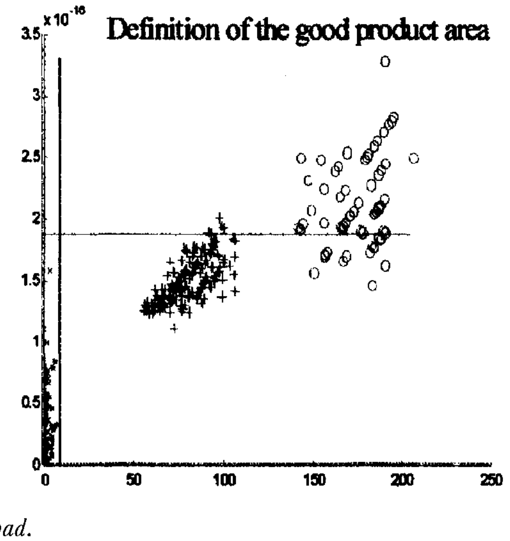

*Good–bad.*

| | Theoretical | Predicted |
|---|---|---|
| A | 0.3 | 0.295 |
| B | 0.3 | 0.298 |
| C | 0.5 | 0.461 |
| D | 0.5 | 0.471 |
| E | 0.6 | 0.608 |
| F | 0.6 | 0.605 |
| G | 0.6 | 0.599 |

*Quantification.*

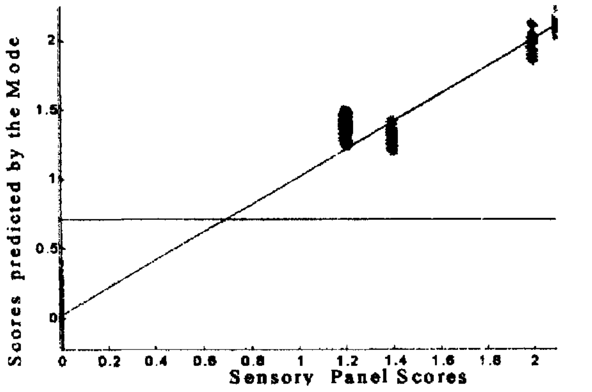

*Sensory panel.*

### *In situ* FTIR for real time monitoring and control of hazardous waste incineration

*Chad M. Nelson and David Marran, Advanced Fuel Research, 87 Church Street, East Hartford, CT 06108-3742, USA*

The USA is interested in developing advanced fossil fuel combustion and waste incineration systems that reduce the emission of harmful species into the environment. The primary hazardous emissions are particulate, unburned hydrocarbons, chlorinated compounds, sulphur oxides, nitrogen oxides and ammonia. An important aspect of most pollution control strategies is the ability to have on-line monitoring of the clean up device. This ensures environmental safety as well as proper operation of the plant. This paper describes the development and testing of second generation Fourier transform infrared-based measurement systems for continuous emission monitoring and process monitoring in high-temperature reaction systems.

A turn-key *in situ* monitoring system was installed at a rotary-kiln, hazardous waste incinerator for more than 2 months, providing continuous concentration data to the plant's distributed control system. Extended testing was performed at a full-scale, rotary-kiln incinerator to rapidly perform *in situ* CO concentration measurements between the rotary-kiln and the secondary combustor using FTIR. The location was chosen for two reasons: (i) it provides the earliest warning possible for potential CO breakthrough; and (ii) once CO is detected, the secondary combustor firing rate can be increased on demand to oxidize the excess CO. Based on the results of this work, the potential for real time combustion control during hazardous waste incineration, based on FTIR analysis has been demonstrated.

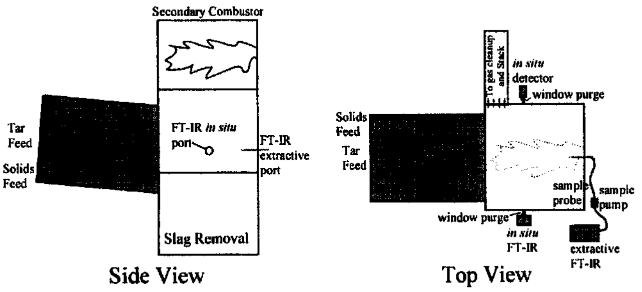

*Schematic diagram of the rotary kiln incinerator showing the* in situ *and extractive monitoring points.*

### Data compression and spectral deconvolution of IR images

*Chris W. Brown, Dongsheng Bu, Scott W. Huffman, Nancy P. Camacho[1] and Richard Mendelsohn[2], Department of Chemistry, University of Rhode Island, Kingston, RI 02881, USA; [1]Hospital for Special Surgery, Research Division, 535 E. 70th Street, New York, NY 10021, USA; [2]Department of Chemistry, Rutgers, The State University of New Jersey, Newark, NJ 07102, USA*

The use of CCD cameras and the recent addition of focal plane array detection systems for measuring the spectra of spatial images has led to a dramatic increase in the amount of spectroscopic data. Instead of a single spectrum from an object, we can obtain spectra from multiple locations on the surface of an object. For example, a camera with a spatial resolution of 600 × 500 pixels will generate 300 000 spectra. To examine all of these spectra is virtually impossible. Even to store and process this much information is a difficult task for most of the present generation of fast, desktop computers with gigabytes of memory. As it turns out, many of the spectra measured at individual pixels are redundant or, at least, linearly dependent upon other spectra or combinations of spectra in the data set. Typically, two-dimensional spatially distributed spectra will contain information on a few chemical components present in the object under investigation. Potentially, the storage requirements and processing times can be greatly reduced through the use of chemometric methods.

The overall goal of this project is to develop and test methodologies for compressing multi-dimensional data sets and to provide the methodology for extracting pure component spectra from the spectrum of a mixture at each pixel. Presently, it is possible to produce false colour maps of a surface by assigning colours to spectral band intensities or band ratios. However, it would be more meaningful to produce false colour maps by assigning colours to the total spectral intensity of individual chemical components. Once this methodology is in place, it will be necessary to store only the component spectra and their relative concentrations at each of the pixels.

A novel method for extracting pure component spectra from mixture spectra will be presented. This method employs principal component analysis (PCA) to reduce the dimensionality of the data. Pure component spectra are then extracted from PCA loading vectors (spectra) using a series of selection rules. The methods for preprocessing and for applying the selection rules will be demonstrated on FTIR image spectra of bone and ligament tissues.

### An instructional software package on FTIR spectrometry

*L. Avila, L. Fine and B. Venkataraman, Department of Chemistry, Columbia University, NY, NY 10027, USA*

We have developed an interactive software package on the theory and practice of FTIR spectrometry. After introducing the fourier transform with illustrations and applications drawn from different fields (e.g. music and medicine), the software turns the user's attention to the question of how the fourier transform and interferometry work together in recording an infrared spectrum. It starts by showing how the Michelson interferometer is used to determine an absorption spectrum with a monochromatic light source, if the sample absorbs at this frequency. From there it expands to a two-frequency light source and then to a polychromatic light source showing how the principles are the same, just that the interferogram becomes more complex. Issues related to resolution and optimum usage of anodization functions are discussed. On the experimental side, trading rules are illustrated along with other issues, e.g. the purpose of the HeNe laser in the instrument. It ends with some applications of FTIR spectrometry.

Where possible, interactive animations have been included to help explain concepts. For example, in a section contrasting a dispersive spectrometer with an FTIR spectrometer, the user 'walks' through the instrument with the components in the instrument 'clickable' to view additional information or simple animations on the purpose and working principle of the component. Each section ends with a quiz to test the users on their understanding of the material presented.

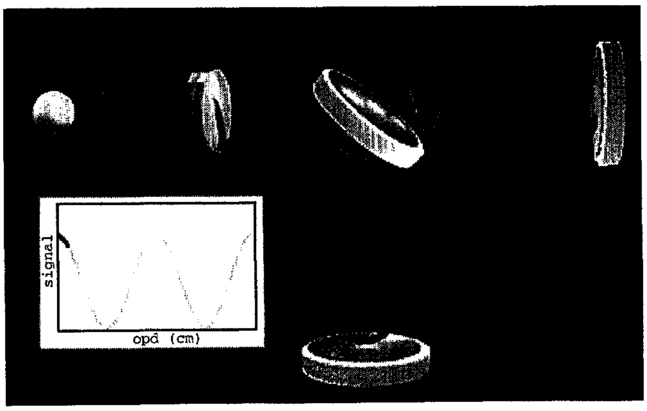

*A screen shot from an animation illustrating how an interferogram is sampled (the light source is monochromatic).*

### Dedicated infrared sensor systems for process monitoring

*Suneet Chadha, William Kyle, Larry Curtiss, Roy Bolduc[1] and Mark Druy[1], Foster-Miller, 195 Bear Hill Road, Waltham, MA 02154, USA; [1]Sensiv, 195 Bear Hill Road, Waltham, MA 02154, USA*

Foster-Miller has leveraged its innovations in infrared fibre optic probes and the recent development of a miniature spectrometer to build a novel infrared sensor system for process applications. A mid-IR fibre optic attenuated total reflectance (ATR) or diffuse reflectance (DRIFT) sensor coupled to a small footprint spectrometer that can detect and quantify organics provides the enabling technology for remote measurement with high detection levels.

The developed sensor system is a low-cost alternative to process IR systems. The ATR element fabricated out chalcogenide fibres enables acquisition of mid-IR spectral data from liquid samples and is sensitive enough to provide detection limits well below 0.5%. The DRIFT probe allows surfaces to be sampled in a non-contact mode. Such sensors coupled to the miniature spectrometer provide significant cost advantage over conventional sampling methodologies.

The miniature spectrometer, which for the intended use, has dimensions of the order of 6″ × 4″ × 2″, can equal the measurement accuracy and throughput of an FTIR. The spectrometer is based on a unique wedge-grating optic that uses low-cost thermopile point detectors to pick off the diffracted wavelengths.

The sensor system eliminates the cost, complexity, reliability and bandwidth/resolution problems associated with either Fabry Perot or Michelson interferometer-based approaches for low-cost process applications. Given the small size and performance insensitivity to vibration, high EMI, thermal variations, the system would easily adapt to remote measurement requirements for process applications.

We will present the enabling innovations along with present performance and sensitivity expectations in this paper.

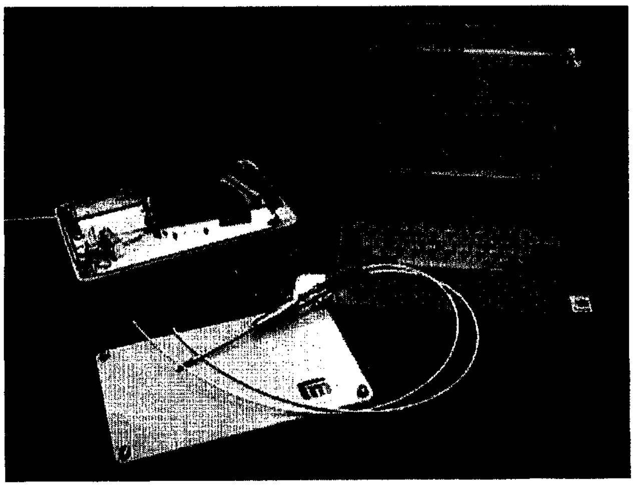

*ATR-probe coupled dedicated IR monitor.*

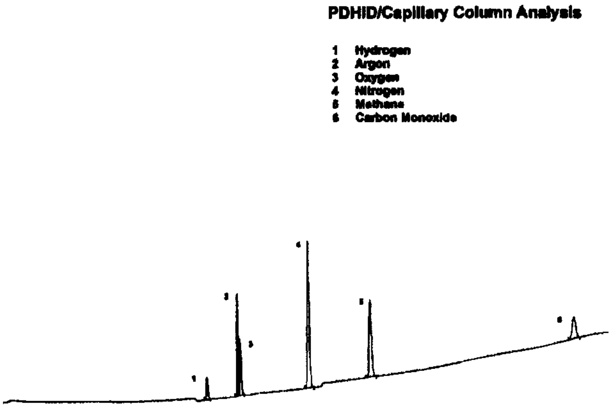

### Turn-key on-line analysis of trace permanent gas impurities by gas chromatography

*David Cuthbert, Wasson-ECE Instrumentation, Ft. Collins, CO 80524, USA*

The semiconductor industry utilizes a wide variety of bulk gases throughout the wafer fabrication process. The processes involved require that these gases be as pure as possible. Some sources of contamination are the permanent gases: hydrogen, argon, oxygen, nitrogen, methane and carbon monoxide. Because of the toxic or pyrophoric nature of many of these chemicals, automated analysis of the impurities would provide the highest levels of safety and reliability. Historically, this has been very difficult to achieve. The discharge ionization detector, required for the analysis of the permanent gases, requires specific engineering for operation in a process environment. In addition, the standard on-line chromatography available on the market today does not have sampling systems that are suitable for handling ultra pure bulk gases.

Working with a variety of gas producers, we have developed an on-line gas chromatograph to analyse for trace permanent gas impurities in bulk gases. The system uses a Hewlett-Packard 6890 gas chromatograph, equipped with a discharge ionization detector. Throughout the unit, high purity parts and orbital welding are used to ensure safety and reliable data. The gas chromatograph is housed in a self-contained NEMA 4 enclosure which is rated for operation in a Class 1, Div 2, Group C or D environment. If the gas being analysed is toxic, a sensor monitors the atmosphere in the enclosure and shuts off the sample supply if a leak is detected.

The gas chromatograph's software controls a sample supply panel, automatically selecting between 11 available process streams. The software directs the necessary valve and instrument control systems as well as providing data handling functions. The results of the analysis are supplied to a remote computer, which trends the data and alerts the operators of any system alarm conditions. Chromatograms and repeatability are shown.

### What cost quality? Accreditation's impact on the quality assurance laboratory

*Douglas Zacherl, Quality Assurance Department, Polymers Division, Bayer Corporation, New Martinsville, WV 26155, USA*

Today's business environment places a great deal of emphasis on laboratory accreditation. The accreditation is seen as a placard of the laboratory's competency. It provides 'proof' that the lab generates quality results and provides quality service.

While the laboratory accreditation process is a means to improve or display the high quality of service provided by the analytical laboratory, it is also a valuable marketing tool for the laboratory and the company. The accreditation is used to both attract new customers and retain present customers who are requiring accreditation from their suppliers. The process, however, provides its benefits at a price. It most definitely adds to the cost of doing business.

There is much more to the cost of the accreditation process than the fee itself. The laboratory must initiate new levels of documentation, mandate the use of NIST traceable standards throughout its system, and convert to accredited and certified vendors where possible. It must conduct instrumentation certification programs, ensure data traceability back to original observations, and institute a system to track the lab's status with regard to each guideline covered by the accreditation. For laboratories that were not required to follow GLP, conforming to these requirements can be a monumental task. Most personnel not directly involved with the accreditation process see only the end result of the process and not this high cost in time and money required by the accreditation.

Once an accreditation is obtained the task then becomes keeping it. The steps taken and changes instituted to attain the accreditation must become the laboratory's SOP if it is to be maintained. The process never really ends. Accreditation is not the end goal, it is the means by which the goal is attained.

### An Internet-accessible database of natural matrix reference materials for environmental and nutritional studies

*Andreas R. Bleise, D. Glavic-Cindro[1], Robert M. Parr, International Atomic Energy Agency (IAEA), Vienna, Austria; [1]Jozef Stefan Institute, Ljubljana, Slovenia*

The International Atomic Energy Agency (IAEA) assists laboratories to demonstrate their ability to perform analytical measurements thereby also giving them the opportunity to improve their analytical performance. Certified reference materials are a major component of analytical quality control systems. Ideally the matrix of the reference material should be as similar as possible to that of the test samples. The IAEA maintains a database which is intended to help laboratories in selecting a suitable reference material by providing information on what is internationally available.

The information included in the database refers to natural matrix reference materials for trace elements, organic constituents, radionuclides, inorganic compounds, extractable elements and organometallic compounds. The database presently contains over 20 000 values for 480 measurands and 1085 reference materials from 43 different producers. The information has been extracted from the relevant certificates of analysis, information sheets and other reports provided by the reference material producers. The matrices are arranged in eight different groups. The matrix classification shows that most of the materials are of non-biological origin (soils and sediments, rocks and geological materials, anthropogenic pollution materials). The measurands are divided into 10 different groups. The five inorganic classifications (trace and macro elements, inorganic compounds, extractable constituents, radionuclides, organo-metallic constituents) are provided in a periodic table, the five organic groups (agrochemical contaminants, aliphatic and aromatic hydrocarbons, chlorinated hydrocarbons, other organic compounds, veterinary drugs and other toxins) are in alphabetical order.

Information included in the database is on values for measurands determined in reference materials, producers, suppliers, on the cost of the materials, the unit size supplied, and recommended minimum weight of material for analysis, if available.

This database is now accessible over the Internet via the IAEA's website. It will help analysts to select reference materials for quality assurance purposes that match as closely as possible, with respect to matrix type and concentrations of the measurands of interest, their samples to be analysed.

### Available cyanide determination using USEPA method OIA 1677

*Michael R. Straka and Liljana Solujic[1], OI Analytical, P.O. Box 9010, College Station, TX 77842-9010, USA; [1]University of Nevada-Reno, Reno, NV, USA*

On 7 July 1998, the USEPA proposed a new distillation-less methodology in the Federal Register for the determination of available cyanide, method OIA 1677. The new method involves gas diffusion flow injection analysis combined with ligand exchange reagent technology and amperometric detection.

This paper will present a complete description of method OIA 1678, the performance that can be expected when using the method, and a description of the lessons learned during the past 5 years of real-world analysis using the method in a wide range of industries and sample matrices.

Additionally, recent research has demonstrated that the method can also be used as the finishing step for the standard distillation procedure, offering many of the same advantages and benefits while allowing solid samples to be distilled directly. Results of a comparative study between the calorimetric and amperometric finishing step will be presented including interference studies, cyanide metal complex recoveries, and analysis of real-world samples.

### Total cyanide determination using USEPA draft method OIA 1678

*Michael R. Straka and Liljana Solujic[1], OI Analytical, P.O. Box 9010, College Station, TX 77842-9010, USA; [1]University of Nevada-Reno, Reno, NV, USA*

With a recent USEPA proposal in the Federal Register, the cyanide-regulated industry is close to gaining access to a novel distillation-less methodology for available cyanide. However, most cyanide permits are written with total cyanide as the regulated species. A method for total cyanide based upon the technology of OIA 1677 would offer the cyanide-regulated community a major advantage over the current 2-h distillation followed by pyridine/barbituric acid calorimetric finish.

This paper will present a complete description of USEPA draft method OIA 1678, total cyanide via gas diffusion flow injection analysis/UV digestion/amperometric detection. It will include metal cyanide species recovery data, interference studies, sample pretreatment procedures, nine laboratory USEPA round-robin data, and method validation criteria that will be used to establish quality control limits.

In addition, a discussion of real-world sample matrix issues will be discussed in this paper.

### Verifying incoming manufacturing ingredients in food products using FT-Raman and FT-NIR

*Michael Longmire and Scot Ellis, Nicolet Instrument Corporation, 5225 Verona Road, Madison, WI 53711, USA*

To assure the quality and safety of their products, food manufacturing companies must verify that the ingredients they use are correct. The verification of incoming ingredients can be performed in many ways, as long as the manufacturer has reasonable assurance that they are correct. Methods used for incoming ingredient verification must be specific and properly validated. This to ensure that the manufacturer and regulatory agencies are satisfied that mistakes have a minimal chance of occurring. Many methods used to verify ingredients require sample preparation and may be time consuming or subjective. Modern spectroscopic techniques exist which can be set up to complete the verification of ingredients very rapidly with minimal or no sample preparation. Fourier transform Raman (FT-Raman) and fourier transform near-infrared (FT-NIR) spectroscopy are two such techniques. These techniques are gaining favour in the food industry as they can complete rapid screening of powders and liquids directly in plastic, glass containers, or directly in large drums.

This paper will compare and contrast the techniques of FT-Raman and FT-NIR used for the identification and verification of ingredients used in the manufacture of food products. Topics that will be discussed include the use of sampling methods with no sample preparation, requirements for building spectral databases, and the use of results in the decision-making process.

### Monitoring the chemical and biological environment of the crew habitat in space

*Darrell L. Jan., Jet Propulsion Laboratory, California Institute of Technology, Pasadena, CA 91109, USA*

Future space missions will require astronauts to live in an isolated, closed environment or habitat for many months at a time. Such a habitat will recycle air and water by chemical or biological means, or some combination thereof. It will be critical to monitor the environment for a number of possible hazards: slow build up of trace chemicals released by any number of mechanisms; accidental release of hazardous chemicals; accidental release of hazardous biological material; or a change in the environmental ecology in a harmful direction.

The NASA Life Sciences Division has established the Advanced Environmental Monitoring and Control (AEMC) Program in order to encourage monitoring and control technologies which can benefit the crew habitat, and which take advantage of the modern techniques for miniaturization, as well as advancements in biotechnology. Because of the constraints of space flight, it is crucial that monitoring technologies be lightweight, small, low in power consumption, and low in maintenance and operation effort.

The AEMC Program has been in place for ~4 years. The program develops technologies through the following means: (i) funding of technology development through a peer-reviewed proposal process; (ii) tracking technology development in related fields and encouraging interactions; (iii) selecting candidate technologies for further ground and space flight development and testing.

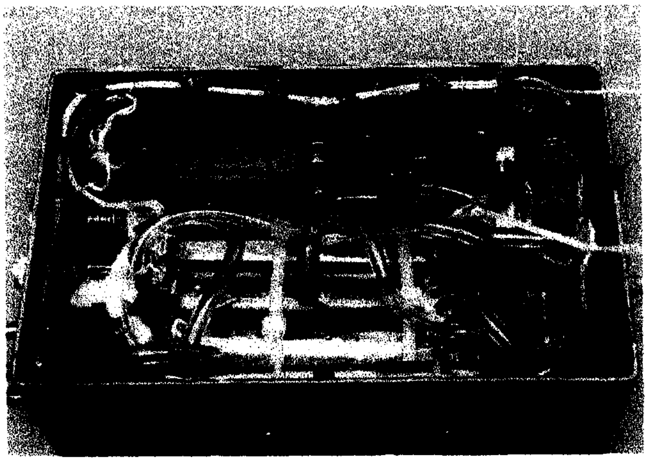

*Interior view of an electronic nose for monitoring spacecraft air.*

### Cognitive systems: physiologically inspired pattern recognition for chemical analysis

*Paul E. Keller, Pacific Northwest National Laboratory, PO Box 999, Richland, WA 99352, USA*

Cognitive systems are emerging information technologies inspired by the qualitative nature of biologically based information processing found in the nervous system, human reasoning and decision-making, and natural selection. Often implemented with artificial neural networks, fuzzy logic and evolutionary computation, these technologies exploit the tolerance for imprecision and uncertainty found in biological systems to achieve tractability, robustness and low-cost solutions to engineering problems.

The electronic nose is a natural match for physiologically motivated chemical analysis. Both the olfactory system and the electronic nose consist of an array of chemical sensing elements and a pattern recognition system. The approach to chemical analysis implemented in an electronic nose is substantially different than that found in conventional analytical chemistry. While analytical chemistry is generally used to precisely identify and quantify concentrations of chemicals, electronic noses are generally used to produce qualitative results indicating the presence of a substance or quality of a product. Thus, electronic noses fill a different niche than traditional analytical chemistry.

This presentation discusses physiologically motivated information processing and how it can be applied to pattern recognition for chemical analysis in electronic

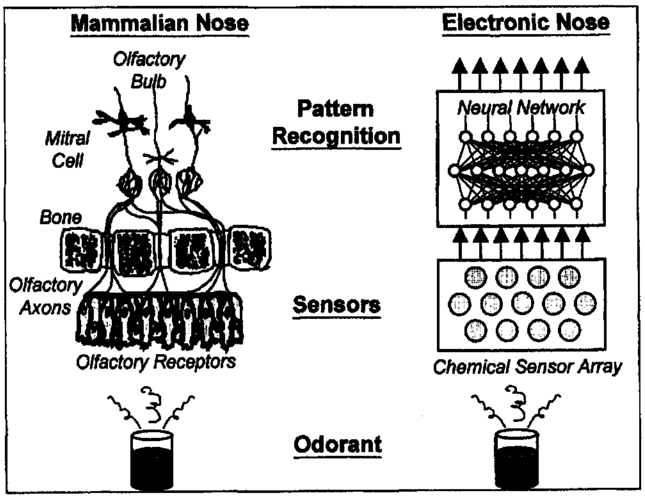

*Basic structures of mammalian and electronic noses.*

noses, and how electronic noses are used in environmental sensing, chemical process control, food processing and medicine.

### A hybrid electronic nose system utilizing a mass spectrum detector and a proprietary array of metal oxide sensors

*John Poling, Quitterie Lucas and Frederic Zenhausern, Alpha M.O.S. America, 102 Towne Centre Drive, Hillsborough, NJ 08876, USA*

Sensor array systems have been effectively used to discriminate samples and correlate a system response to a meaningful description of a product's quality. The goal in configuring these devises has been to select sensing devises that provide good selectivity, sensitivity and stability, and design systems which can provide the information at a QC or shop floor level where the maximum benefit can result.

This paper will provide an overview of new advances in sensor system technology. The first of these advances is a hybrid detection system consisting of two different types of gas sensors: a mass spectrometer (MS) detector combined with a proprietary array of metal oxide sensors (MOS). By combining these two complimentary techniques, the issues of selectivity (MS) and sensitivity (MOS) of the measurement can be optimized.

Also introduced at this time will be a technique for rapid headspace generation through microwave heating of the sample. By using this novel headspace generation method, it is possible to decrease the time in which the headspace of a sample can be generated and improve the overall efficiency of the generation. Data will be presented demonstrating improvement in the VOC yields of up to 90% with generation time reduced by 30 s to 2 min.

Results will be presented utilizing both of the sensor technologies citing applications of foods, flavours and chemicals for both qualitative and quantitative analytical problems. These applications were carried out to address a variety of different aspects of the samples investigating such things as contamination, intensity of flavour, and aging or spoilage of the samples.

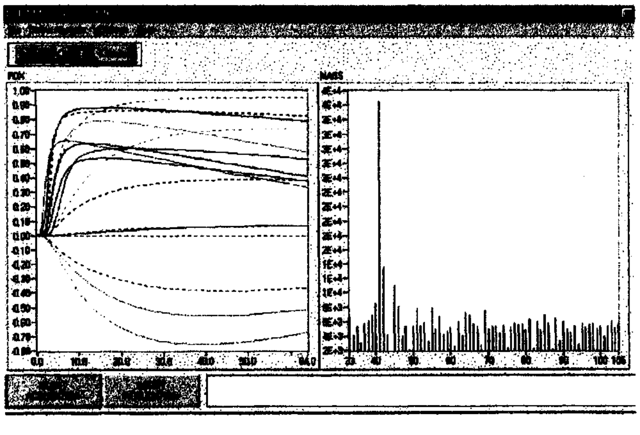

### Strategies for improving odour analysis using a GC-based electronic nose

*Omowunmi A. Sadik, Miriam Masila and Marc Breimer, Department of Chemistry, State University of New York at Binghamton, P.O. Box 6016, Binghamton, NY 13902-6016, USA*

In recent years, some instrumental odour analyses utilizing a combination of headspace sampling, non-specific chemical sensor arrays and pattern recognition techniques have been described. The instrument is suitable for different applications, including food, clinical and environmental measurements, and is commonly referred to as an electronic nose (EN) or electronic olfactometer. EN sensors use either metal oxides, quartz crystal arrays, surface acoustic wave devices, electrochemical cells or conducting polymers, or even a combination of these sensors for mimicking the human sense of smell.

In addition, EN instruments use either equilibrium or dynamic stripping sampling techniques. In each case, an unknown quantity of air (or vapour) sample from the headspace is drawn through the sensor surface for analysis. EN techniques are generally good for qualitative analysis but unsuitable for quantitative analysis. The limitations include short-term sensor drift and mass-transfer limited signal reproducibility at the sensor–analyte interface. Recently, we have used the concept of gas chromatography coupled with electronic nose to introduce a known quantity of the sample into the system for analysis. The new GC-based EN technique was used to determine low-vapour odorants, including polyaromatic hydrocarbons, industrial solvents and polychlorinated phenols. This approach did not only yield better sample handling, but also provided a way of separating samples containing more than one component.

This paper describes the strategies for complete odour analysis, including identification, correlation and source recognition by determining the relationships between odours and their activities. Also, we describe the study of sensor-array surfaces under well-defined conditions and examine alternative approaches to achieving better distinction of the parameters that influence the properties of odours or tastes.

### Improved automatic sample preparation for regular, ultra-low-carbon and high-alloyed steel

*Gunther Hawickhorst and Theodore Kurela[1], Herzog Maschinenfabrik GmbH & Co., D49086 Osnabruck, Auf dem Gehren 1, Germany; [1]Herzog Automation Corp., 16600 Sprague Road, Cleveland, OH, USA*

The constantly increasing demand by the steel industry for more accurate analytical results forces all leading suppliers of sample preparation machines to improve existing designs and develop new machines.

In most steel mills, samples are prepared either with a combination of a coarse and fine abrasive belt, a combination of a cup wheel and a fine belt, or just with a single belt or single cup wheel. Invariably, small amounts of burnt binder or belt material will contaminate the surface of the sample, influencing the analytical results, particularly for samples where carbon has to be determined in the ultra-low range.

To overcome this problem, Herzog has developed a new fully automatic sample preparation machine, Herzog Model HB4000. This machine uses a wide coarse belt for the preliminary surface preparation. The final surface preparation is accomplished with either a wide fine belt or a milling head. Milling of the sample for the final surface preparation will produce uncontaminated surfaces for ultra-low-carbon samples being analysed on optical emission spectrometers.

The use of the milling head or the wide fine belt for the final surface preparation step is also being evaluated for high-alloyed steels, which are typically analysed on X-ray spectrometers where the results on certain elements are significantly influenced by variations in sample surface.

This machine was developed by using proven components from other existing Herzog preparation machines, has been tested since July 1998, and initial operating results will be reported in this paper.

### Identification and quantification of organic contaminants on silicon wafer surfaces using thermal desorption GC-MS/AED

*Peng Sun, Marty Adams and Terrie Bridges, MEMC Electronic Materials, 501 Pearl Drive, St. Peters, MO 63376, USA*

Organic contaminants on silicon wafer surfaces can have detrimental impacts on the fabrication of semiconductor devices. Major impacts include haze degradation, lower breakdown voltage, surface hydrophobicity, post-CVD defects, non-uniform CVD film thickness, unintentional doping and formation of silicon carbide. Advances in the electronic industry toward large-scale integration of semiconductor devices have placed strict demands on the ability to measure and monitor ultra-trace levels of organic impurities on silicon wafer surfaces. This paper describes a thermal desorption gas chromatography-mass spectrometry/atomic emission detection (TD-GC-MS/AED) method for ultra-trace analysis of organic contaminants on silicon wafer surfaces. Organic contaminants on wafer samples are thermally desorbed from the surface and transferred to a sample collection tube which is packed with graphitized carbon material. The sample collection tube is then loaded onto an automated thermal desorption sample introduction unit coupled to a GC instrument. Following GC separation, column effluent is split to a mass spectrometer (MS) for unknown identification and to an atomic emission detector (AED) for elemental analysis. This on-line combination of MS and AED provides a powerful tool for simultaneous identification and quantification of surface organic contaminants. Total organic carbon, phosphorus, sulphur and other elements of interest, as well as structural information of organic contaminants, are obtained in a single GC-MS/AED run. The advantages, method accuracy, reproducibility, detection capability, and application of this technique will be discussed in further detail in this paper.

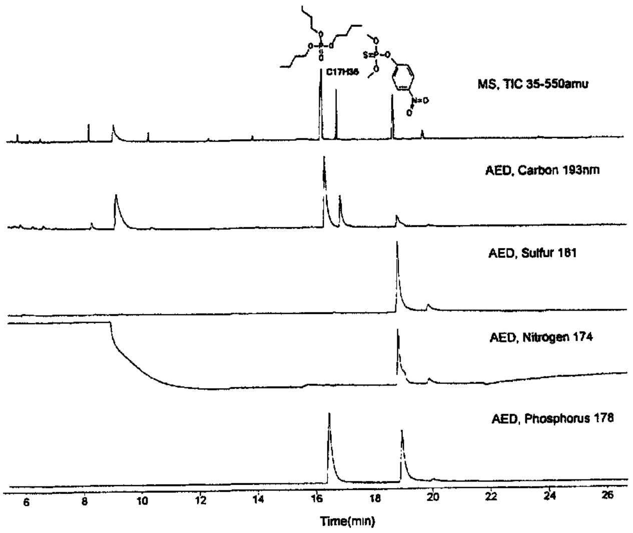

### Improvement of reliability in ICP-MS by multivariate calibration in the case of seawater analysis

*K. Danzer, I. Thielen, C. Fischbacher and M. Paul*[1]*, Department of Analytical Chemistry, University of Jena, Lessingstr. 8, D-07743 Jena, Germany;* [1]*Perkin-Elmer Germany, Askaniaweg 1, D-88662 Überlingen, Germany*

Multivariate calibration (MVC) is widely used in spectroscopy when unspecific signals have to be evaluated, as is the case in NIR- and UV-VIS spectrometry. But MVC can also be applied successfully in optical emission spectral analysis and ICP mass spectrometry where well-separated and specific signals predominantly appear. In such cases, MVC application effects an increase of sensitivity and also a decrease in limit of detection. Moreover, a considerable part of the disturbed signals can be included in the evaluation. This fact directed the attention to ICP-MS which is affected by serious background and signal interferences by molecule ions and isotopes. In analytical practice, there are several possibilities to overcome such interferences, mainly the use of high-resolution instruments or coupling with separation techniques. Another way is the correction of interferences by mathematical equations which are limited in application. Analyses of alloys have shown that multivariate calibration can successfully be used in ICP-MS. The precision and accuracy could be significantly improved in this way.

Seawater is a difficult matrix system for ICP-MS because of serious interferences by the main components. The trace analysis of heavy metals, e.g. V, Cr, Ni, Fe, Cu, Zn, Co and Ti is not only disturbed by argon-containing molecule ions but also by high concentrations of interfering components, e.g. Na, Cl and Ca ($CaO^+$). It will be shown that the reliability of trace analyses in seawater can be improved significantly by application of MVC. By comparison with correction procedures, it will be shown that MVC is applicable more in general and without any pre-information on the interfering species because disturbed signals can be included into the PLS evaluation. Using MVC it is also possible to extract relevant signal information from disturbed signals and to relate this with the corresponding analyte concentration.

So, MVC should be an interesting alternative to the application of high-resolution ICP mass spectrometry.

### The determination of trace metals in seawater using ICP-MS

*Steven M. Wilbur, Hewlett-Packard, Chemical Analysis Group, 15815 SE 37th St., Bellevue, WA 98006, USA*

Analysis of trace metals in seawater by ICP-MS presents a number of interesting challenges. These challenges are due to the typically low level (ppt to low ppb) of most trace metals in ambient seawater and the high concentration of matrix elements, e.g. Na, K, Ca, Mg and C. These matrix elements can contribute to significant spectroscopic and non-spectroscopic interferences which, if uncorrected, can result in unacceptably high detection limits for many analyte elements. While no single technique is optimum for all analytes and conditions, some strategies for reducing or eliminating interferences include the use of elemental equations, hydride generation, matrix elimination, standard additions, shield torch techniques and others. Various techniques for optimizing and automating the analysis of trace metals in seawater will be discussed, e.g. the use of a shield torch with hot plasma for the analysis of As, $^{80}$Se and Hg by hydride/cold vapour ICP-MS. Results of the analysis of standard reference materials and low-level matrix spikes will be presented in this paper.

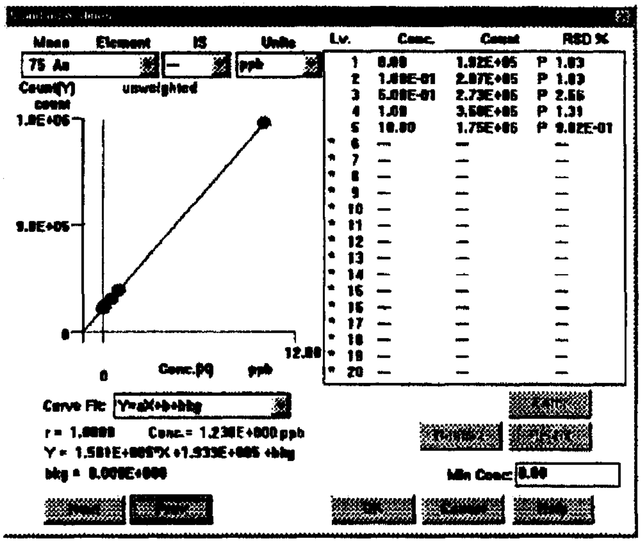

*Arsenic in undiluted seawater by on-line hydride shieldtorch ICP-MS (standard addition calibration). Calibration range: 0.1–10 ppb.*

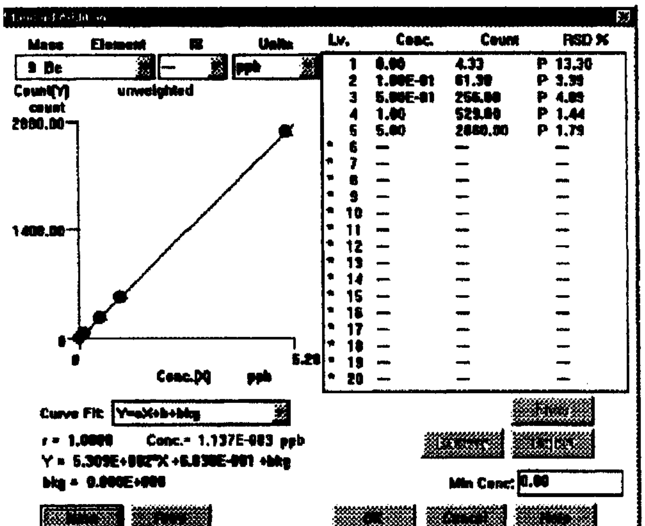

*Beryllium in 1.5 diluted seawater by direct injection ICP-MS (standard addition calibration). Calibration range: 0.1–5.0 ppb.*

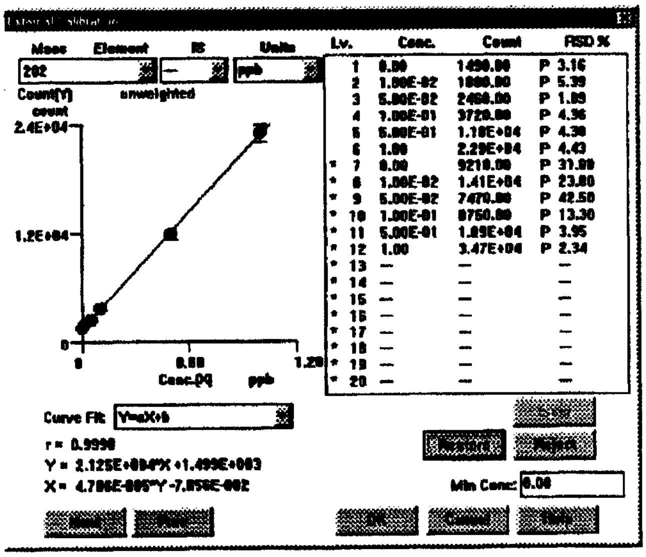

*Mercury in undiluted seawater by on-line cold vapour shieldtorch ICP-MS. Calibration range: 0.01–1.0 ppb.*

### Automated speciation of arsenic compounds in food composites by ion-exchange chromatography-inductively coupled plasma mass spectrometry (IEC-ICPMS)

*Olujide T. Akinbo, Reshan A. Fernando, James H. Raymer and Edo D. Pellizzari, Research Triangle Institute, Analytical and Chemical Sciences, 3040 Cornwallis Rd., Research Triangle Park, NC 27709-2194, USA*

Ion exchange chromatography-inductively coupled plasma mass spectrometry (IEC-ICPMS) is one of the most used analytical techniques in environmental speciation studies of metals and organometallic compounds. However, due to software and hardware incompatibilities between most ICPMS and ion exchange chromatographs, laboratories using these techniques must perform their analyses manually. This presents economic disadvantages and reproducibility problems. In our case, automation of the operation of an IEC-ICPMS system was accomplished by using the Gilson control language code resident on a VG Plasma Quad ICPMS to initiate sample injection by a Waters 717 liquid chromatography autosampler. The autosampler in turn initiates the chromatographic program on a Waters 626 liquid chromatography pump. This system was used to evaluate the performance of a method developed for the simultaneous speciation of both cationic and anionic arsenic species on an anion exchange column. The arsenic speciation method is based on various extraction schemes. The automation procedure, extraction efficiencies of the extraction schemes, and overall analytical performance of the method (linearity range, detection limit, precision and accuracy), will be discussed in this paper.

### As(III) analysis using a flow injection hydride generation ICP-MS (FIAS-HG-ICP-MS)

*Xiaoshan Chen, Metropolitan Water District of Southern California, 700 Moreno Ave., La Verne, CA 91750, USA*

An analytical method using a flow injection hydride generation-inductively coupled plasma-mass spectrometry (FIAS-HG-ICP-MS) was developed for arsenite [As(III)] analysis. The method uses a Perkin-Elmer FIAS-400 flow injection analysis system (FIAS) coupled with an ELAN 6000 ICP-MS. Inorganic As(III) is selectively converted on-line to arsine ($AsH_3$) using sodium borohydride and l-cysteine/nitric acid. The arsenic hydride is purged with argon into ICP-MS for As detection.

Compared to the previous hydride generation method, this method provides the following advantages. First, it combines the low detection limit capability of ICP-MS and small sample volume and high precision of FIAS to provide a method with low detection limit and high reproducibility. Secondly, it is highly selective to inorganic As(III), providing an As(III) speciation capability. Thirdly, it does not use HCl acid, and therefore greatly reduces or even eliminates chloride inferences for the ICP-MS. Fourthly, the method uses low $HNO_3$ concentration (less than 1% $HNO_3$ is used in l-cysteine solution), which helps reduce the amount of $H_2$ generated, resulting in a more stable plasma and improved precision. In addition, this method requires minimal sample preparation, and uses only one tubing size for all on-line reagent additions, simplifying hydride generation operation and optimization.

This method has been shown to be highly selective for As(III), and therefore is suitable for speciation analysis of arsenic (III). Other forms of arsenic have been shown to be unable to generate arsine under the conditions in this method, Tested arsenic species included inorganic arsenite [As(III)], inorganic arsenate [As(V)], monomethylarsonic acid (MMA) and dimethylarsinic acid (DMA). The detection limit for As(III) was found to be <0.01 μg/l.

### A universal and fast GC method for the analysis of aromatic hydrocarbons in the process laboratory

*James D. McCurry, Hewlett-Packard, Wilmington, DE 19808, USA*

A number of different ASTM D-16 methods have been developed for the analysis of impurities in aromatic hydrocarbons. These methods are commonly used in the petrochemical industry to test feedstocks and final products as well as monitor processes. However, maintaining many separate methods can be complex and time consuming.

This paper will present a simple and rugged GC method that can be used for the analysis of many materials usually performed with 10 separate ASTM D-16 methods. The technique of retention time locking (RTL) was used with this method to minimize retention time differences between GC systems. This allows easier method maintenance and simplified comparison of results. Method translation software was used to convert this method for use with shorter and narrower columns to accommodate faster analysis in process control applications. Retention time and quantitative reproducibility will be discussed along with decreases in analysis times for typical aromatic hydrocarbon samples.

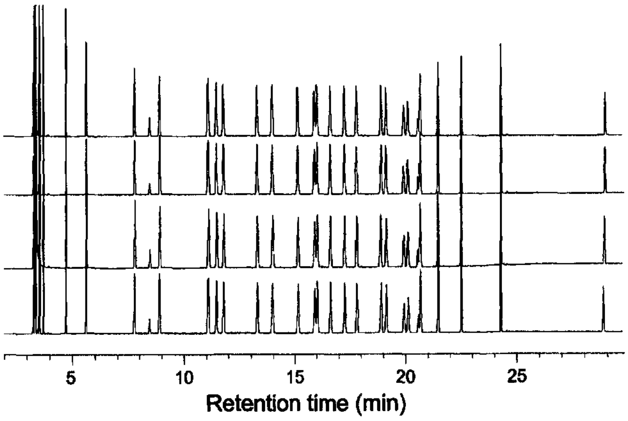

*Separation of 28 compounds typically found as impurities in many aromatic hydrocarbons. Retention time locking was used to get nearly identical retention times for all compounds on four different GC systems.*

### Design and performance of a multi-valve system in process gas chromatography

*Yang Xu and Teresa J. Lechner-Fish, Daniel Industries, 9753 Pinelake Drive, Houston, TX 77055, USA*

The properties of the process sample, the composition of the sample during upset and the operation in hazardous locations affect the analytical method development and the hardware design. Because process gas chromatographs are installed at the process to analyse the sample continuously, the hardware must be designed for reliable analyses and low maintenance.

To provide a reliable process gas chromatograph, the valve system should have the flexibility for multiple analyses (multiple valves and detectors). In addition, the valve design must be leak free and have a quick response. Because the gas chromatograph system can be contaminated during upset conditions, the valve system should be easily cleaned or replaced.

To optimize the chromatography, the valve system should be heated independently of the columns. The temperature should be precisely controlled in a comprehensive oven system, which includes insulation, sensor, heater and temperature controller. If packaged properly, the thermal mass of the valve system can greatly improve the temperature stability of the system (patent pending).

In this paper, system design, operating characteristics and performance of a multi-valve system will be discussed. In addition, repeatability data for a light hydrocarbons ($C_1$–$C_{10}$) application will be presented.

### On-line HPLC analysis of alkyl phenols

*John Wasson and Richard Tuley*[1]*, Wasson-ECE Instrumentaion, Ft. Collins, CO 80524, USA;* [1]*Schenectady International, Freeport, TX 77541, USA*

A specific product in a chemical plant is a complex mixture of alkylated phenols. This mixture, when analysed by a gas chromatograph with a flame ionization detector, yields a chromatogram with a multiplicity of isomers and unwanted by-product contaminates. A lack of detector discrimination makes analytical results tenuous.

Product quality assessment requires the determination of the *o*-dodecyl phenol to *p*-dodecyl phenol ratio. As identification of these species is difficult by gas chromatography, other technologies were investigated. A better solution was found to be the employment of a high-performance liquid chromatograph (HPLC) equipped with an ultraviolet detector. The ultraviolet detector could analyse for the phenol chromophore, while ignoring extraneous sample components.

The instrument used for this analysis was a Hewlett-Packard 1100 HPLC. For feedback and control, the instru-ment discussed was converted for on-line use in a hazard-ous environment. This allowed for the fastest analytical results, while reducing the manpower requirement.

Placing the instrument Class 1, Div. 2, groups C & D environment required the development of a rugged sample system. Special engineering was also required to handle the highly viscous sample and flammable mobile phase. Analytical capabilities and aspects of conversion for on-line use, are detailed.

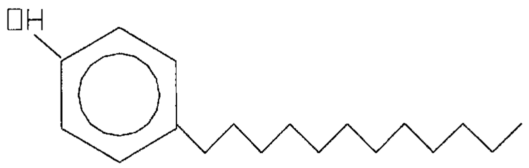

p-*dodecyl phenol.*

### A new system for on-line analysis of fluorinated by-products in LPG process streams

*Michael L. Duffy, OI Analytical, P.O. Box 9010, College Station, TX 77842-9010, USA*

In many areas, the petrochemical industry residual by-products created in the refining process can cause degradation of pipelines, machinery and many facets of processes 'downstream' of particular processes. Residual fluoride is one such by-product that can rapidly degrade or destroy transport devices, but more importantly it can destroy many of the catalytic beds used in specific refinery processes, resulting in millions of dollars of repair and replacement costs.

The common method historically used for fluoride testing is ASTM Method D2784, commonly referred to as the 'Wickbold' method. This method involves safety-related issues due to the use of a $H_2/O_2$ burner, and takes ~3 h to perform. Reliability in the low ppm range is only fair and ppb measurements are not feasible.

For over 10 years, OI Analytical has sold electrolytic conductivity detectors (ELCDs) as an alternative to the Wickbold method. As this alternative test method began to grow in use and popularity, OI Analytical began to offer complete, turn-key laboratory-based GC systems used in many petrochemical plant QA/QC laboratories to perform this now preferred alternative method.

With this increased use came requests to design a similar system to that used in the lab but configured as a process analyser that could be installed directly on-line in the refinery (Class 1, DIV. II area). This system permits continuous analysis on a 24-h basis, on multiple (eight) sample streams. This paper describes this new system, the OI Analytical fluorinated by-products analyser (FBA), and reports on the results obtained following the installation of two units at one location.

### Optimization of parameters involved in sensors for simultaneous ratiometric fluorescence measurement of oxygen and carbon dioxide

*Sunil Dourado and Raoul Kopelman, Department of Chemistry, University of Michigan, Ann Arbor, MI 48109-1055, USA*

Oxygen and carbon dioxide monitoring is directly related to metabolism, hence is extremely important in biological systems. They are important parameters in the diagnosis and treatment of critically ill patients as well as in environmental monitoring and process control. Sensors aimed at detecting $O_2$ and $CO_2$ simultaneously allow one probe to measure their concentrations. Oxygen- and carbon dioxide-sensitive luminescence indicators and reference dyes have been incorporated into a polymeric membrane for their detection by ratiometric fluorescence measurements.

The polymer membranes are made of silicones, varieties of polyurethane, polystyrene and sol-gels. Oxygen is measured by observing fluorescence quenching of a suitable dye. Ruthenium, palladium and platinum dyes are used as oxygen-sensitive dyes. Carbon dioxide is monitored by tracking the change in the fluorescence emission spectrum of a suitable pH indicator dye as the gas diffuses through the polymer membrane and interacts with the dye. Indicator dyes studied include HPTS and CNF. Reference dyes are chosen such that they are unresponsive to both oxygen and carbon dioxide.

The dual sensor is composed of a polymer matrix containing the three dyes needed to simultaneously detect $O_2$ and $CO_2$, using a ratiometric fluorescence technique. Data will be provided for sensors using different combinations of dye and polymer. Sensor response to oxygen and carbon dioxide in aqueous

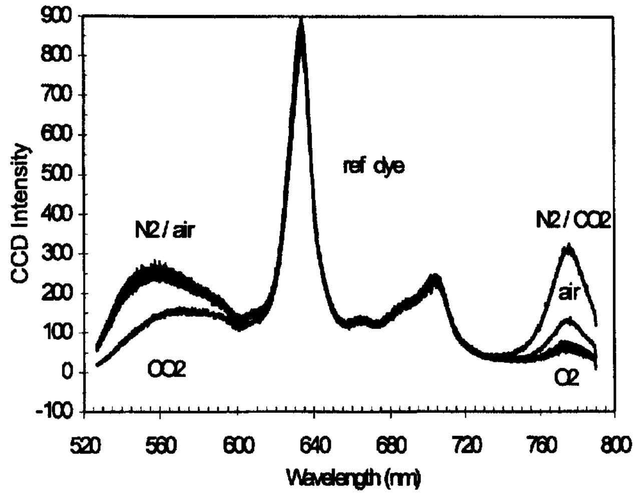

*Fluorescence emission spectrum of a polystyrene dual gas sensor under the influence of air, nitrogen, oxygen and/or carbon dioxide. The dyes used were HPTS (550 nm), OEP (632 nm and 705 nm) and PtOEPk (775 nm).*

solution will be elucidated. Parameters that will be optimized are leaching, photobleaching and response time.

### A new forensics technique to investigate the presence of chemical 'fingerprints' in human breath

*Daniel B. Cardin and Michael R. Winward, Entech Instruments, 2265A Ward Ave., Simi Valley, CA 93065, USA*

The determination of whether an individual has been in a particular building or location has involved the collection of physical evidence, including hair, fingerprints, and items left or removed by the individual under investigation. Chemical testing of body fluids and cell DNA has also been used to effectively show whether an individual has been at a particular place, if such pieces of evidence can be recovered. Many times, no such evidence is found making it difficult to place an individual at the scene of a crime.

A new technique is being investigated that takes advantage of the somewhat unique 'chemical fingerprint' found inside each building. This fingerprint is the result of the summation of organics slowly outgassing from hundreds of different sources within a particular building, generally making the distribution of these chemicals different between different buildings. In some cases, e.g. illegal drug manufacturing laboratories, concentrations of organic vapours can be substantially elevated providing a very distinct chemical fingerprint relative to typical indoor air. When inhaled, these chemicals can absorb through the pulmonary alveolar membrane in the lungs into the blood stream where they can later re-exchange back into exhaled breath over the course of several days. Small, fused-silica-lined stainless steel sampling canisters have been used to successfully collect samples from human breath for determination of chemicals at concentrations up to hundreds of times lower than those found in typical indoor air. The determination is made by automated preconcentration of the sample by a factor of 1000 to 1 000 000 and then injecting the concentrated organics into a trace level GCMS system for quantification. Estimations of chemical longevity in the human body can be evaluated using test subjects whose exhaled breath can be compared to that of an individual under investigation after having been exposed to the air in a building for a similar period of time.

### Preparing difficult forensic samples for automated solid phase extraction

*Andrea Belec and Lynn Jordan, Zymark Corporation, 68 Elm Street, Hopkinton, MA 01748, USA*

The use of solid phase extraction (SPE) in forensic toxicology drug confirmations is continually increasing. The small sample size required by SPE is conducive to the limited amount often available to the toxicologist. Demanding caseload schedules are driving laboratories toward automation in the hope of improving speed of extraction, productivity and safety. Automating confirmations also reduces employee exposure to potentially harmful samples.

This paper will present several models of pre-extraction sample preparation techniques utilizing different dilution reagents, concentrations and precipitations. Extractions were performed using the RapidTrace SPE Workstation followed by GC/MS analysis. Several different brands of SPE cartridges were evaluated to determine differences in flow characteristics, sample handling and recovery.

### Automated DNA typing: high-throughput sample processing and objective DNA profile designation for forensic investigation

*Stewart M. Allen, South African Police Service, Forensic Science Laboratory, Private Bag X620, Pretoria, 0001, South Africa*

A computer program has been developed for automated deoxyribonucleic acid (DNA) length polymorphism result designation and sample process management. Criminal casework requires efficient recording of exhibits, while DNA analysis guidelines require among other things, duplication and separation of control samples from exhibit samples. These processes can be efficiently managed according to user requirements. Sample sheets for the Apple Macintosh-based applied biosystems (AB) sequencers are automatically generated, preventing typing mistakes and minimizing sample switches.

Raw data, generated by AB automated DNA sequencers, are imported and results designated. The system, called STRgazer due to the use of simple tandem repeats (STRs) in current DNA typing methods, is part of a larger laboratory information management system (LIMS), called STRlab.

The STRlab system is not limited to any specific commercial DNA typing kit and allows simultaneous use of different DNA typing kits. A complete log of sample process, run results, including all non-allelic peaks, is maintained such that all sample history can be easily queried.

An integrated DNA profiles database, called STRbase, stores DNA profiles determined by STRgazer. STRbase is independent of the number and type of loci attached to a sample. Additional loci results can also be added indefinitely to any sample. STRbase can also store partial and mixture profiles as well as being able to import any external DNA profile data, including HLADQAI, DI S80 or any other non-PCR single locus profile data. A matching profiles search engine module, STRquest is also built in and is capable of searches including partial profiles and mixture results.

The STRlab LIMS satisfies the requirements as specified by TWGDAM, EDNAP and ASCLD for forensic DNA typing as well as providing an objective alternative to manual typing methods.

User and sample type definable security access control also allow this program to be used for general casework DNA analysis methods or for criminal intelligence data banking.

### Continuous *in vivo* monitoring of glucose with subcutaneously implanted miniature wired enzyme electrodes

*Adam Heller and David W. Schmidtke, Department of Chemical Engineering and Texas Materials Institute, The University of Texas at Austin, MC C0400, Austin, TX 78712-1062, USA*

The glucose concentrations in the subcutaneous interstitial fluid of rats and in a brittle juvenile diabetic chimpanzee were monitored amperometrically with a miniature (0.3 mm diameter, $5 \times 10^{-4}$ cm$^2$ area) electrode on which glucose was directly electro-oxidized.

In the chimpanzee, the output of the electrode was monitored by a wrist-worn potentiostat. The microelectrode tracked the glucose concentrations through the physiological range encountered in diabetes (2–30 mM) after its one-point *in vivo* calibration. The correlation coefficient ($r^2$) between the subcutaneous interstitial fluid and the capillary blood glucose concentrations was 0.94, and regression analysis (at 95% confidence interval) yielded the relationship capillary blood glucose = 0.98 ($\pm 0.05$) subcutaneous glucose concentration 4.2 ($\pm 12.6$ mg/dl). One-point *in vivo* calibration was practical even during periods of rise or decline, e.g. following insulin injection.

Monitoring of glycaemia in the non-diabetic rat following intravascular insulin injection revealed a $24.5 \pm 6.8$ min lag time between the intravascular and the subcutaneous hypoglycaemic nadirs, and resulted in a transient maximal difference averaging $30.7 \pm 12\%$. The transient difference was mathematically modelled, and algorithms were developed allowing prediction of the venous blood glucose concentrations from the subcutaneous measurements, and prediction of the subcutaneous fluid glucose concentrations from the intravenous measurements following insulin injection.

The $5 \times 10^{-4}$ cm$^2$ mass-transporting area of the microsensors was substantially smaller than that of previously reported glucose-sensing microelectrodes. Reduction of the mass-transporting area was made feasible by the direct transduction of the glucose concentration into an electrical current. Earlier *in vivo* sensors were usually based on reacting glucose with dissolved oxygen, the concentration of which in the interstitial fluid is less than 0.2 mM, 150 times less than glucose concentration in a hyperglycaemic patient, and the current density was defined by the oxygen permeating area, which was at least 30 times larger than the area required for glucose permeation. To avoid oxygen consumption, the enzyme, glucose oxidase, was directly 'wired' to the electrode through an electron-conducting and glucose-permeable redox hydrogel.

### Monitoring multi-route exposures of children to pesticides in the home environment

*Robert G. Lewis, National Exposure Research Laboratory, US Environmental Protection Agency, Research Triangle Park, NC 27711-2055, USA*

Pesticides, whether used inside the home or outside on the lawn and garden, accumulate on indoor surfaces, especially in carpet dust, and also in upholstery and in or on children's toys. Semivolatile pesticides exchange between surfaces and air, thereby contributing to increased indoor air levels. For example, pesticides sprayed on the lawn are tracked indoors, where they are protected from environmental breakdown and can persist for months or years, as opposed to perhaps days outside on the grass. Typically, pesticide concentrations in indoor air and house dust are 10–100 times those found in outdoor air and surface soil. Because small children spend most of their time indoors, much of this time in contact with floors, and engage in frequent mouthing of hands, toys and other objects, they are the population at highest risk for exposure. Whereas acute exposures of small children to freshly-applied pesticide residues warrants considerable concern, chronic exposure to pesticides in house dust may present a larger potential problem.

Methodology for measurement of most pesticides in air is generally good and a good database exists for indoor air levels of many pesticides. On the other hand, methods for assessment of the relative importance of dermal contact exposure to and incidental (non-dietary) oral ingestion of household pesticides are lacking. Better tools for measurement of pesticides in house dust and for determination of surface-dislodgeable residues are needed, along with methods for estimating dermal exposure, especially for infants and toddlers.

Over the past several years, monitoring devices for measurement of the dislodgeability of pesticide residues from residential surfaces have been designed, tested and standardized through ASTM. Methods have also been developed to study the movement and distribution of pesticides around the household, as well as the potential routes of children's exposures to them. The transfer efficiencies of pesticide residues from surfaces to dry and saliva-moistened hands have been determined. House dust has been separated into seven size fractions ranging from <3.5 μm to >250 μm, and each fraction analysed for a broad spectrum of pesticides. Controlled or simulated studies have been carried out to determine transfer of particles from smooth and soft (carpeted) surfaces to skin, the movement of soil from outdoors to indoors, and the re-entrainment of particles from the floor into the air as the result of human activity in order to obtain exposure estimates. In addition, studies have been conducted in occupied residences to determine the intrusion of pesticides applied to lawns into the home and their redistribution between surfaces and air indoors. Activity patterns of children must also be studied to assess the nature and frequency of their contact with pesticide residues.

Once there are accurate means to determine what is picked up on the skin, further research will be necessary to estimate what is removed by mouthing and absorbed through the skin. These studies are currently underway. Exposures through the dermal contact route must then be aggregated with inhalation and dietary exposures to determine total exposure as mandated by the Food Quality Protection Act of 1996.

### Determination of nitrogen by flow injection analysis in environmental and wastewaters

*Kathleen A. Straw and Evangeline M. Hodge, Los Alamos National Laboratory, CST-9 Trace Inorganic Analysis Group, MS E-518, Los Alamos, NM 87545, USA*

Flow injection methodology (FIA), as a versatile tool for nitrogen analysis in a wide variety of water samples, is presented. Dilute radioactive liquid waste generated from the labs, accelerators, reactors and shops at LANL and directed to a central chemical wastewater treatment plant make up a large portion of our routine workload. In addition to these treatment–process samples, other environmental surveillance, NPDES, R & D and spill-

CALIBRATION AND SAMPLE PEAKS TKN 660nm:

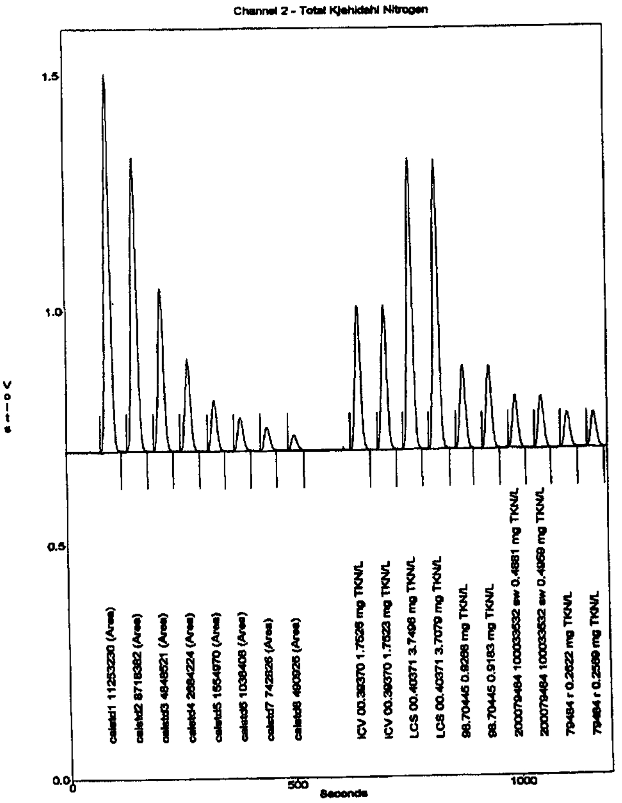

identification samples are analysed using two FIA systems. EPA-approved UV/VIS methods used include ammonia–nitrogen (phenolate), nitrite–nitrogen (diazo calorimetric), nitrate–nitrogen (diazo with cadmium reduction), and total Kjeldahl nitrogen (salicylate–nitroprusside calorimetric).

Work on several research studies for reduction of nitrates in LANL's radioactive liquid waste treatment plant effluent is summarized: (i) bacterial reduction of nitrates to nitrogen gas as employed by sanitary sewage treatment plants; and (ii) electrolytic reduction of nitrates to other nitrogen species more easily removed from dilute radioactive liquid waste.

### Automated SPE techniques for handling dirty wastewater samples

*Bob Johnson and Kathy Pappas, Horizon Technology, 8 Commerce Drive Atkinson, NH 03811, USA*

Today's environmental laboratories face three main challenges: improving productivity; providing accurate, defensible data in a timely manner; and minimizing the cost of analysis. Sample preparation is generally the most problem-prone and often the most expensive step in the analytical laboratory.

Laboratories are placing an increasing emphasis on using new technology and automation to improve productivity. Solid phase extraction (SPE) disk technology has clearly demonstrated several advantages over classical liquid–liquid extraction (LLE). However, SPE techniques are used predominately for the analysis of drinking water. To apply SPE technology for the analysis of many SW 846 methods requires the ability to handle dirty samples. Dirty samples often have a high level of particulate matter that can clog SPE disks. Techniques for processing aqueous samples with high particulate content will be addressed.

SPE uses less solvent than traditional LLE and eliminates emulsions. Automated SPE, using the SPE-DEX® system from Horizon Technology increases productivity and consistency of the extraction. The SPE-DEX® system performs the extraction with minimal operator set up.

This presentation will focus on the benefits of automating the SPE procedure and approaches to adapting SPE for traditional LLE methods. Strategies for processing aqueous samples with high particulate contents using automated SPE will be discussed.

### Validation of electronic nose technology for food ingredients

*Joost Vanhemelrijck and Peter De Cock, EBS Food Ingredients, Eridania Beghin-Say, Vilvoorde Research and Development Centre, Havenstraat 84, B-1800 Vilvoorde, Belgium*

A number of electronic noses are now available to the food industry. After a thorough evaluation, the eNOSE 5000 from EEV Chemical Sensor Systems (formerly Neotronics Scientific) was selected for purchase. The initial aim was to investigate the potential of the eNOSE 5000 to assess odour/taste quality of various food ingredients produced by Eridania Beghin-Say (EBS). It is EBS

policy to keep up to date with the latest analytical technology.

At the EBS Research and Development Centre, Vilvoorde, Belgium, the eNOSE 5000 has been evaluated as a complementary tool to expensive and subjective sensory panels, as well as GCMS, for the assessment of the quality of a variety of EBS products. The ultimate goal is to investigate the potential of this new technology for plant production control of large numbers of production batches.

For each type of food ingredient, a thorough methods development study has been performed. This includes the effect of sample variables, sample preparation techniques, choice of sensor type, analytical procedures, etc. The emphasis has been focussed on reproducibility of sensor responses and validity of multivariate statistical techniques.

Food ingredients examined include carbohydrates, oils, lecithins and proteins. The results clearly indicate that the eNOSE 5000 is able to differentiate between sensory qualities of many food ingredients, and as such can be a powerful tool to support traditional human sensory panels in the food industry. However, it should be remembered that electronic nose technology is relatively new and will probably undergo many changes in the future.

### The response of the human nose and other instruments to petrochemical taint in seafood

*F. Aladar Bencsath, James D. Barnett[1], Roberta G. Berg[2] and John D. Poling[3]; Food and Drug Administration, Gulf Coast Seafood Laboratory, Dauphin Island, AL 36528, USA; [1]Food and Drug Administration, Seattle West Coast Laboratory, Bothell, WA 98041, USA; [2]Food and Drug Administration, Southeast Regional Laboratory, Atlanta, GA 30309, USA; [3]Alpha M.O.S., Hillsborough, NJ 08876, USA*

Oil spills contaminate fishing waters, seafood and sealife. The mandatory closure of the affected waters is lifted by the authorities only after seafood is proven to be acceptable by organoleptic assessment and chemical analysis. Organoleptic assessment is carried out by a panel of trained analysts using a 'passed' or 'failed' designation. Proper analytical instruments could provide objective, reproducible and more detailed characterization of taint and natural odour components. GC/MS combined with dynamic headspace sample enrichment technique (HS/GC/MS) is probably the most sensitive and accurate method for such analyses, but is too tedious for instant field-use and larger series of samples.

'Electronic nose' instruments (from AromaScan, Neotronix and Alpha M.O.S.) record and store 'aroma patterns' without chromatographic separation. We carried out exploratory measurements on untainted fish and fish tainted at several levels with crude oil and petroleum products using a few of these instruments. HS/GC/MS and organoleptic assessments were made for comparison. We found that commercial instruments equipped with arrays of metal oxide sensors (MOS) were more responsive to petroleum taint than were those of conductive polymer sensors. One MOS-based instrument was sensitive enough to detect oil traces in fish at the 5 ppm level, which is the lower threshold concentration for the human nose to recognize crude oil in fish. HS/GC/MS conveniently detected and identified a series of hydrocarbon compounds in fish tainted at that level.

### Chemical vapour comparisons using a hand-held SAW microsensor array

*Hank Wohltjen, Norman O. Davis, Brent Busey, Mark Klusty, Robert Saling and Harold McKee, Microsensor Systems, 62 Corporate Ct., Bowling Green, KY 42103, USA*

The rapid detection and identification of chemical vapours in the field is an increasingly important analytical requirement. We have developed a powerful hand-held instrument that uses an array of polymer-coated surface acoustic wave (SAW) resonators to provide 'fingerprint' patterns for a variety of organic compounds ranging from simple solvents to complex mixtures (e.g. perfumes, fuels, etc.). These digitized patterns can be readily compared with patterns previously stored in the instrument library. The complete system weighs ~1 lb and includes a small sampling pump, SAW microsensor array, data acquisition and pattern recognition computer, liquid crystal display, and rechargeable battery pack.

The resulting instrument has been used successfully as a 'vapour comparator' to identify chemical vapours by determining the relative partition coefficient of a vapour

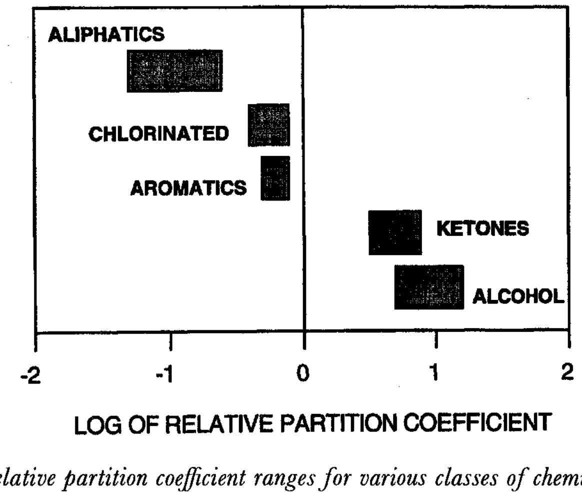

*Relative partition coefficient ranges for various classes of chemical vapours.*

and finding the closest match to values stored in its library. The relative partition coefficient values are stable with time and permit the discrimination of such things as fuel type (e.g. gasoline versus diesel) and octane rating (e.g. 87 versus 93). Analysis times (including sampling, detection and pattern recognition) as short as 20 s have been demonstrated.

### Simultaneous detection of enantiomers using amperometric biosensors in flow injection systems

*Jacobus F. Van Staden, Raluca-Ioana Stefan, Hassan Y. Aboul-Enein[1] and George-Emil Baiulescu[2], Department of Chemistry, University of Pretoria, Pretoria 0002, South Africa; [1]Bioanalytical and Drug Development Laboratory. Biological and Medical Research (MBC-03), King Faisal Specialist Hospital and Research Centre, P.O. Box 3354, Riyadh 11211, Saudi Arabia; [2]Department of Analytical Chemistry, Faculty of Chemistry, University of Bucharest, Blvd. Regina Elisabeta # 4-12, 703461 Bucharest-3, Romania*

Enantioselective analysis has become increasingly important in the analysis of pharmaceutical products, because many of the drugs marketed today are administered as racemic mixtures despite the significant differences in pharmacological, pharmacodynamics and pharmacokinetics of individual enantiomers.

Amperometric biosensors assured for enantiomers assay the best enantioselectivity and sensitivity.

Their response characteristics [large working concentration range, low detection limits ($10^{-6}$–$10^{-12}$ mol/l)] as well as the reliability of the analytical information obtained by using these amperometric biosensors as detection systems made them suitable as detection devices for flow injection analysis (FIA).

The simultaneous detection was possible by incorporating the biosensors in series into the flow system. The accuracy of the analytical information as well as the rapidity (more than 65 samples/h) of simultaneous detection is better than in the chromatographic methods, due to the possibility to monitor directly, without any prior separation, both enantiomers in solution.

The advantages of using amperometric biosensors for simultaneous detection of enantiomers will be highlighted for L- and D-amino acid oxidase-based biosensors used for simultaneous detection of some angiotensine-converting enzymes (ACE) inhibitors enantiomers.

### Environmental monitoring of organic vapours by polymer-coated thickness-shear mode quartz resonators

*Radislav A. Potyrailo, Timothy M. Sivavec, Edward B. Stokes, Patricia D. Mackenzie and Joseph J. Salvo, General Electric Company, Corporate Research and Development, Schenectady, NY 12301, USA*

Piezoelectric devices are increasingly being studied as detectors for chemical species. These instruments have the high potential to achieve detection limits of real-time measurements at parts-per-billion levels at a low cost and in a portable configuration.

With the goal for applying these sensors for environmental monitoring needs, we have developed a prototype instrument for the real-time detection of low concentrations of organic vapours. Our chemical sensor is based on a thickness-shear mode (TSM) 10 MHz AT-cut quartz resonator coated with a non-polar polymer film. The resonator is powered by an oscillator circuit at its fundamental frequency. The output frequency of the oscillator is measured by a frequency counter and then recorded and analysed on a laptop computer.

Upon exposure to trichloroethylene (TCE) vapour, the operating frequency of the polymer-coated TSM resonator linearly decreases as a function of the increasing mass of the sorbed vapour in the polymer film. The sensor response is completely reversible with the 1-min response and recovery times. The sensor demonstrates the detection limit of 1 ppm of TCE vapour when measurements are performed at 22 °C with referencing to a frequency of the sensor in a blank gas. Arranging the sensor in the headspace above water can lead to the determination of TCE in ground- and drinking water. The 1 ppm detection limit of our sensor for TCE in air corresponds to 15 ppb TCE concentration in water, according to Henry's law. This is at least an order of magnitude improvement over other reported 10 MHz TSM-based sensors.

In addition to the discussion of sensor performance, we will analyse the factors needed to produce a polymer film useful for stable operation over an extended period of time. Also, several environmental parameters including ambient temperature and relative humidity will be evaluated as contributors to the sensor instability and film aging.

### Determination of Se in urine by flow injection hydride generation electrothermal AAS with on-line microwave digestion

*Julian Tyson, Robert Ellis and Pablo Carrero, Susan McIntosh[1] and Frank Fernandez[1], Department of Chemistry, Box 34510, University of Massachusetts, Amherst, MA 01003-4510, USA; [1]Perkin-Elmer Corporation, 761 Main Avenue, Norwalk, CT 06859, USA*

The determination of total selenium in human urine is a non-trivial analytical problem. Although concentrations are within the capabilities of electrothermal AAS, it is not possible to find furnace operating conditions in which all the various forms of selenium are reliably converted to one precursor prior to atomization and there is a major spectral interference from phosphate-containing compounds. In principle, hydride generation provides the means to overcome matrix interferences, but it is necessary to convert all species to selenium(IV). This can be done in a batch procedure in which the sample is digested under reflux with a mixture of bromate and hydrobromic acid. However, this procedure is relatively slow, and an improvement in performance characteristics would result from the development of an on-line version of the procedure. Initial manifold designs, in which the sample merged with reagents and then passed through a microwave field in a 'focused' reactor device, were only partially successful; not all species were converted to

Se(IV). The most difficult compound to convert is trimethylselenonium (TMSe), known to be a major metabolite in human urine. Complete destruction of the TMSe was only obtained when the manifold was reconfigured so that the sample and reagents were trapped under stopped flow conditions in the microwave field under elevated pressure. An automated FI procedure was constructed around an eight-port two-position rotary valve. To 1 ml of urine was added 10 ml of concentrated HBr and 0.5 ml of 0.7 M $KBrO_3$, and, after dilution to 25 ml, a 500-μl subsample was injected into the manifold. It was not necessary to add hydroxylamine hydrochloride to destroy excess bromine. The working range was from the detection limit of 0.2 μg/l to 80 μg/l and the precisions at 10 and 30 μg/l were 3 and 2% RSD, respectively. Complete recoveries of 20 μg/l Se spiked into urine were obtained for selenocystine, selenomethionine, selenoethionine and TMSe.

### Preparative countercurrent chromatography with a coaxial rotating system

*Yue-Wei Lee, Du Qi-Zhen*[1] *and Scott Raleigh*[1]*, Chemistry and Life Sciences, Research Triangle Institute, Research Triangle Park, NC 27709-2194, USA;* [1]*Natural Pharmacia International, Research Triangle Park, NC 27709-2882, USA*

Countercurrent chromatography offers distinct advantages in preparative purification of water-soluble natural products. It is a continuous liquid–liquid partitioning process that separates a mixture by their differential volubility in two immiscible solvents. Recent advances have enabled the countercurrent chromatography to be operated in a high-speed manner. It has become an important complementary technique to HPLC in natural product research. However, there are limitations in scale-up purification (>10 g) because of excessive centrifugal forces generated in a large centrifuge, and the capital costs of building such a large system are very expensive.

For preparative purification of water-soluble natural products, a coaxial rotating system has been evaluated in our laboratory. Several parameters, e.g. flow rate, rotational speed and stationary phase retention are investigated using natural products or synthetic intermediates. We concluded that the coaxial rotating system can be developed into a highly cost-effective and environmentally friendly method for large scale (>10 g) separation. The results of several large-scale separations will be presented.

### Large-scale preparative purification of crude extract from natural products and synthetic intermediates

*F. E. Chou, Y. Ma*[1]*, W. Li*[2]*, H. Y. Guh*[2] *and Y. Ito*[3]*, PharmaTech Research Corporation, 6807 York Road, Baltimore, MD 21212, USA;* [1]*Laboratory of Ocular Therapeutics, National Eye Institute, NIH, Bethesda, MD 20892, USA;* [2]*The W.R. Johnson, Pharmaceutical Research Institute, Johnson and Johnson, Spring House, PA 19477, USA;* [3]*Laboratory of Biophysical Chemistry, National Heart, Lung, and Blood Institute, National Institutes of Health, Bethesda, MD 20892, USA*

Critical evaluation of potential application of countercurrent chromatography (CCC) for industrial processing of fermentation materials and crude extracts from phytochemical materials prompts us to investigate the scale-up parameters of pH-zone-refining CCC. Based on our laboratory experimental results we were able to process 20–30 g of crude extracts from natural products by using an 850-ml capacity column within 5 h.

The selection of solvent systems and pH condition were repeated to ensure the reproducibility which will be used for scaling-up by using even larger capacity CCC instruments (1500–3000 ml). Our aim is to be able to process up to 100 g of samples, which is ideal for pilot plant projects in an industrial setting.

Three examples of separations by regular and pH-zone-refining CCC will be presented and discussed in this paper.

(1) Isolation of trace impurities from synthetic anticancer drug, cladribine, for the treatment of hairy cell leukaemia and multiple sclerosis.
(2) Purification of anti-HIV lignans.
(3) Separation of 2- and 6-nitro-4-chloro-3-methoxybenzoic acids.

